# Small Molecule‐Templated DNA Hydrogel with Record Stiffness Integrates and Releases DNA Nanostructures and Gene Silencing Nucleic Acids

**DOI:** 10.1002/advs.202205713

**Published:** 2023-02-08

**Authors:** Christophe Lachance‐Brais, Mostafa Rammal, Jathavan Asohan, Adam Katolik, Xin Luo, Daniel Saliba, Antranik Jonderian, Masad J. Damha, Matthew J. Harrington, Hanadi F. Sleiman

**Affiliations:** ^1^ Department of Chemistry McGill University 801 Sherbrooke St W Montreal H3A 0B8 Canada

**Keywords:** antisense oligonucleotides, cyanuric acid, DNA, DNA nanotechnology, gene silencing, hydrogels, stimuli responsive, supramolecular

## Abstract

Deoxyribonucleic acid (DNA) hydrogels are a unique class of programmable, biocompatible materials able to respond to complex stimuli, making them valuable in drug delivery, analyte detection, cell growth, and shape‐memory materials. However, unmodified DNA hydrogels in the literature are very soft, rarely reaching a storage modulus of 10^3^ Pa, and they lack functionality, limiting their applications. Here, a DNA/small‐molecule motif to create stiff hydrogels from unmodified DNA, reaching 10^5^ Pa in storage modulus is used. The motif consists of an interaction between polyadenine and cyanuric acid—which has 3‐thymine like faces—into multimicrometer supramolecular fibers. The mechanical properties of these hydrogels are readily tuned, they are self‐healing and thixotropic. They integrate a high density of small, nontoxic molecules, and are functionalized simply by varying the molecule sidechain. They respond to three independent stimuli, including a small molecule stimulus. These stimuli are used to integrate and release DNA wireframe and DNA origami nanostructures within the hydrogel. The hydrogel is applied as an injectable delivery vector, releasing an antisense oligonucleotide in cells, and increasing its gene silencing efficacy. This work provides tunable, stimuli‐responsive, exceptionally stiff all‐DNA hydrogels from simple sequences, extending these materials’ capabilities.

## Graphical abstract

DNA hydrogels are created which reach record stiffnesses 2 orders of magnitude higher than previously reported. Key to these hydrogels is a novel interaction between polyadenine and cyanuric acid, a mimic of thymine, which forms elongated fibers. These injectable hydrogels are used to release DNA nanostructures and a nucleic acid therapeutic strand, the latter which silences a gene in vitro.

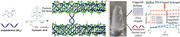

## 1 Introduction

Combining the processability of solids with the diffusion‐enabled reactions of liquids, hydrogels have generated considerable interest for their use in drug delivery, soft‐robotics, analyte detection, cell‐growth, and smart materials.^[^ ^1^ ^]^ Of these, nucleic acid hydrogels—hydrogels formed by supramolecular interactions between nucleic acid strands—present numerous valuable characteristics. The biocompatibility and therapeutic uses of nucleic acids make their hydrogels especially well‐suited for biological applications, such as drug delivery,^[^ ^2^ ^]^ vaccine formulations,^[^ ^3^ ^]^ gene‐activated matrices,^[^ ^4^ ^]^ cellular growth, and tissue engineering.^[^ ^5^ ^]^ Beyond these classical applications, the sequence control of deoxynucleic acids (DNA) hydrogels endows them with unique properties. From selective control of cargo diffusion,^[^ ^6^ ^]^ sequence‐specific changes in moduli,^[^ ^7^ ^]^ chemical dynamic networks,^[^ ^7^, ^8^ ^]^ and tunable sensing of mechanical forces,^[^ ^9^ ^]^ the programmable nature of nucleic acids results in hydrogels with exceptionally precise and complex stimuli‐responsive behavior.

For all their advantages however, DNA hydrogels have issues that prevent their wider applications. First of these is the low mechanical stiffness of unmodified DNA hydrogels, with reported storage modulus (*G*’) from 10^1^ to 10^3^ Pa, with the recent 4 × 10^3^ Pa at 3.5 weigth % (wt%) obtained by the Liu group considered high in the field.^[^ ^10^ ^]^ This low range of stiffness reduces their effectiveness in applications such as soft robotics, and precludes them from some stiffer cell environments such as the spleen.^[^ ^11^ ^]^ DNA hydrogels also suffer from the lack of a convenient chemical handle, preventing the incorporation of useful chemical modifications. Current methods to increase the accessible chemical space rely on costly and low‐density synthetic sequence modifications.^[^ ^12^ ^]^

**Figure 1:**
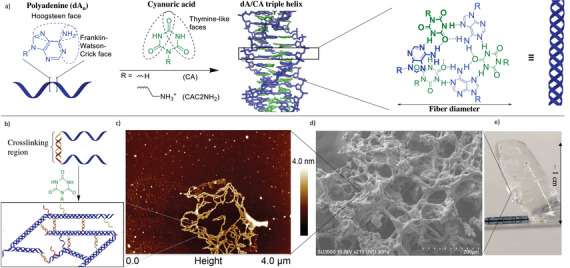
Representative structure of the DNA hydrogels: a) Assembly of polyadenine DNA (dA*_n_*) with cyanuric acid (CA) to form a continuous hydrogen‐bonded helicene structure (right), bringing together a phase‐shifted triple helix that elongates into dA/CA supramolecular fibers. b) Hairy fibers of dA/CA motif crosslinking through their DNA overhangs. c) AFM image of diluted hydrogel of dA_30_‐dsDNA_20_ showing the multimicrometer crosslinked fibers as the basis for the hydrogel assembly. d) SEM image of the freeze‐dried hydrogel showing the fiber bundles and pore structure. e) Image of a hydrogel of dA_30_‐dsDNA_20_ 4 wt% (2 mm, CA 60 mm, Mag buffer) standing at an angle on a razor blade.

Our laboratory has recently discovered a novel noncanonical nucleic acid structure resulting from the interaction of polydeoxyadenine (dA) with the small nontoxic molecule cyanuric acid (CA)—the dA/CA motif (**Figure** **1**a).^[^ ^13^ ^]^ With three faces mimicking thymine, cyanuric acid acts as a bridge between adenines of three poly‐dA strands (Figure 1a), coaxing them into a supramolecular polymer. The polymer is a parallel DNA triple helix formed by a continuous cyanuric acid‐adenine hydrogen‐bonded helical structure.^[^ ^14^ ^]^ Already an unusual DNA secondary structure based on its incorporation of small molecules at high density, this motif also distinguishes itself by its spontaneous formation of multimicrometer fibers which bundle together (Figure 1c). We hypothesized that these fibers could serve as a basis for stronger DNA hydrogels, closer to fibrous protein‐based hydrogels, with a high density of functional groups stemming from the cyanuric acid and its ethylamine sidechain.

Using this dA/CA motif, we here report a hydrogel built from unmodified DNA which has a storage modulus of 10^5^ Pa at only 4.7 w%, which is two orders of magnitude higher than the stiffest reported DNA‐based hydrogels, and which extends the modulus range of unmodified DNA hydrogels to 5 orders of magnitude. It can even conserve a very high modulus at 85 °C. Key to navigating this space is the modularity of the system, in which either complementary overhangs of DNA or cyanuric acid sidechains can control the modulus. This hybrid DNA/small‐molecule hydrogel also naturally exhibits sought‐after properties, such as high functional group density, thixotropy, fast self‐healing, and facile preparation. Our hydrogel also showcases multistimuli responsiveness, including a small‐molecule stimulus rare in DNA hydrogels, while conserving the stimuli responsiveness to specific DNA sequences. We show how these properties can be used to encapsulate and controllably release DNA wireframe and origami nanostructures. We conclude by leveraging stimuli‐responsiveness and thixotropy to make an injectable therapeutic hydrogel that enhances gene silencing activity in vitro.

## 2 Results and Discussion

### 2.1 Tunability of Mechanical Properties

The hydrogels are formed readily by mixing the appropriate poly‐dA containing strands with a solution containing cyanuric acid (CA) or its derivative (CAC2NH_2_) at a slightly acidic pH of 6, then heating at 85  °C and cooling quickly to 20  °C for homogeneous, clear gel formation (Figure 1e) (see the Experimental Section). Usually, the stoichiometry of dA:CA is kept at 1:1—the canonical ratio in the polyA/CA structure—resulting in 20 mm of CA for a 30‐mer of polyA at 700 µm. With this simple assembly, extensive tuning of the resulting mechanical properties is possible by varying the nature of the components.

#### 2.1.1 Polyadenine Length

**Figure 2:**
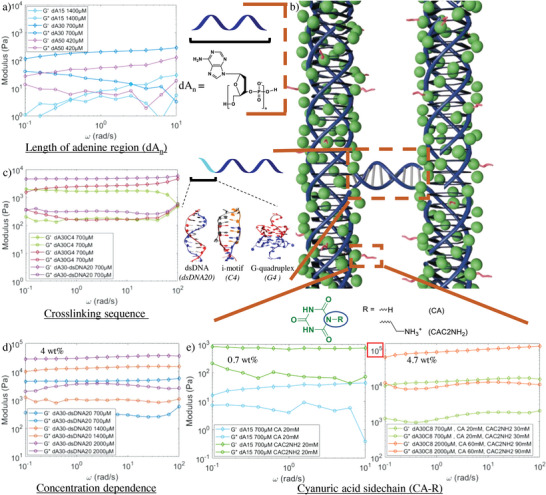
Different parameters tune the mechanical properties of dA/CA hydrogels. a) Rheology of the variation length of the polyadenine region (dA_15_ (1400 µm), dA_30_ (700 µm), dA_50_ (420 µm), all CA 20 mm (dA:CA 1:1)). b) Schematic representation of the crosslinked dA/CA fiber structure with the blue wires representing DNA strands, the green spheres representing cyanuric acid (CA) and the red tails representing its possible sidechain. c) Rheology of crosslinking sequences (all 700 µm DNA and CA 20 mm): left double‐stranded DNA; middle i‐motif, through the assembly the assembly of d(cytosine)_4_ or d(cytosine)_8_ overhangs; right G‐quadruplex through the assembly of d(guanine)_4_ or d(guanine)_8_ overhangs. d) Rheology of DNA concentration dependency for dA_30_‐dsDNA_20_ (CA 20, 40, and 60 mm). e) Effect of the nature of the sidechain of the cyanuric acid: CA and/or CAC2NH_2_ for dA_15_ (left) and dA_30_C_8_ (right). Single replicates are shown for clarity, for other replicates see Figures S11–S20 (Supporting Information).

The first modular component in the design space of our hydrogel is the polyadenine region itself (**Figure** **2**a), which binds to CA, resulting in the dA/CA fibers. The simple addition of CA to dA_15_ gives rise to hydrogels, likely because of fiber entanglement^[^ ^13^, ^15^ ^]^ Changing the length of the dA*_n_* region affects the mechanical properties in a nonlinear fashion. At the same weight % (wt%), the extension of the polyadenine strand from a 15‐mer to a 30‐mer strengthens the storage modulus (*G*’) from 1.3 ± 0.8 × 10^1^ to 1.8 ± 0.4 × 10^2^ Pa at 1 Hz. This likely stems from the tendency of dA_30_ to form more crosslinked networks of fibers compared to dA_15_ as shown previously.^[^ ^15^ ^]^ Upon elongation to a dA_50_ the storage modulus decreases to 4 ± 2 × 10^1^ Pa at 1 Hz, possibly a consequence of the strands being long enough to curl back and copolymerize intramolecularly, rather than with other strands. Moving forward, we thus focused on gels with dA_30_ strands because of their greater mechanical stiffness.

#### 2.1.2 Crosslinking Sequence

To introduce crosslinking between the dA/CA fibers and thus enhance the gel's mechanical properties, we added an overhang region at the end of the poly‐dA strands. Overhang sequences that are capable of interacting with each other through DNA‐based interactions mediate polyA/CA fiber crosslinking. These crosslinks improve the moduli of hydrogels versus uncrosslinked ones by an order of magnitude. For example, strands consisting of a stretch of 30 adenine followed by 20 complementary base pairs as an overhang (dA_30_‐dsDNA_20_) form double‐stranded DNA crosslinks which increase the storage modulus *G*’ (1 Hz) to 4 ± 0.8 × 10^3^ Pa at 700 µm (1.4 wt%) compared to a *G*’ of 1.8 ± 0.4 × 10^2^ Pa for a sequence having simply the dA_30_ region at the same molar concentration (Figure 2a,c,d). At 4 wt% (CA included), *G*’ of these dA_30_‐dsDNA_20_ hydrogels reaches 3×10^4^ Pa, values well outside those reported for previous unmodified DNA hydrogels.^[^ ^9^, ^10^ ^]^ The modulus are now more similar to conventional polymer hydrogels, with our 1.4 w% hydrogel being equivalent to an 8% poly(acrylamide) gel with 1.8% *N*,*N*’‐methylenebis(acrylamide) crosslinker synthesized at 20 °C.^[^ ^16^ ^]^ Due to the predictability of DNA self‐assembly from its sequence, it was possible to design the crosslinks with different motifs, such as the regular double‐stranded helix (dsDNA_13_, dsDNA_20_), the i‐motif (C_4_, C_8_) or the G‐quadruplex (G_4_, G_8_) (Figure 2c), the formation of which is supported by circular dichroism spectroscopy (CD) (Figure S2, Supporting Information). We did not notice large differences between the different lengths of the crosslinking regions or crosslinking type, even if there is an increase in mean modulus in the order i‐motif < G‐quadruplex < double‐stranded DNA (Figure 2c) it is within statistical uncertainty (Figures S11–S20, Supporting Information). While more experiments are needed to elucidate the structural basis of such high moduli, atomic force microscopy (AFM) and scanning electron microscopy (SEM) imaging suggests the material easily forms hierarchical bundles reminiscent of the strong fibers in protein hydrogels (Figure 1d–e).^[^ ^17^ ^]^ The ability to easily control crosslinking from the sequence of DNA provides the material with a second convenient handle to tune the mechanical properties, and can push those surprisingly high for DNA hydrogels.

#### 2.1.3 Cyanuric Acid Sidechain

The third modular handle on our hydrogel is the sidechain of the small molecule cyanuric acid (Figure 2d). We previously demonstrated that CA only needs 2 faces to bind adenine, leaving the third face to display a sidechain onto the triplex exterior.^[^ ^14a^ ^]^ In this previous work, we observed that the substitution pattern on this outward face strongly affected assembly of the fibers, both energetically and structurally. We thus hypothesized that sidechains could have a strong effect on the mechanical properties of the hydrogels formed by these fibers. Substituting CA for its short tailed amino derivative CAC2NH_2_, which we have previously shown to stiffen the motif, increased the storage modulus of pure dA_15_ hydrogels by two orders of magnitude from 10^1^ to 10^3^ Pa at only 0.7 wt% (including CAC2NH_2_), confirming the important role the sidechain has on assembly (Figure 2e, left; and Figure S17, Supporting Information). The stiffness of these hydrogels at such low wt% is closer to the peptide‐based hydrogels rather than conventional DNA hydrogels.^[^ ^17^, ^18^ ^]^ We believe that many factors contribute to this large increase. First, the CAC2NH_2_ carries a positive charge in these conditions, reducing the electrostatic repulsion between the negatively charged DNA fibers and favoring bundling between strands. In addition, we have shown using molecular dynamics simulations that the CAC2NH_2_ derivative reduces motion in the dA/CA motif, possibly leading to stiffer fibers.^[^ ^14a^ ^]^ The CAC2NH_2_ derivative does however have a slightly lower affinity to poly‐dA than its parent compound, probably leading to more vacancies in the triplex at the same concentration and working against its otherwise strengthening effect.

By combining the above lessons on the different handles of our hydrogels, we made a stiff hydrogel with a poly‐dA length of 30 nucleotides, crosslinked by an i‐motif (dA_30_C_8_) in a mixture of CA and CAC2NH_2_. Joining all these handles together, the resulting hydrogel has a *G*’ in the range of 10^4^Pa at only 1.7 wt% (Figure 2e, right). At 4.7 wt% (CA‐R included), it has a *G*’ of 8 ± 1 × 10^4^ Pa at 1 Hz, and 1 ± 0.1 × 10^5^ Pa at 10 Hz. To our knowledge, this is the highest storage modulus of any hydrogel made from unmodified DNA.^[^ ^2^, ^7^, ^10^, ^19^ ^]^ It even surpasses many hydrogels incorporating modified DNA, double‐networks or other alterations to make the materials stronger.^[^ ^20^ ^]^ It also possesses a high thermal stability, the 1.7 wt% gel keeping its moduli virtually unchanged at 37 ˚C (Figure S9, Supporting Information) and the 4.7 wt% gel retaining a storage modulus above 10^4^ Pa even at 85 °C (Figure S10, Supporting Information), and has qualitatively been observed staying a gel at 100 °C.

Taken together, these independent handles allow tuning the mechanical properties of the hydrogel over a large *G*’ window from 10^1^ Pa (i.e., dA_50_ 420µm, CA 20 mm) to 10^5^ Pa (i.e., dA_30_C_8_ 2 mm, CA 60 mm, CAC2NH_2_ 90 mm) by using the base‐pairing properties of DNA combined with the unique nature of the small molecule‐mediated dA/CA motif.

### 2.2 Self‐Healing and Thixotropy

An important property in supramolecular polymers is their ability to self‐heal. Self‐healing is especially important for hydrogels, as it allows them to heal regions broken by cell migration^[^ ^21^ ^]^ or to adapt their shapes as they are inserted or injected in their desired site of interest for drug delivery^[^ ^22^ ^]^ or 3D printing.^[^ ^23^ ^]^

**Figure 3:**
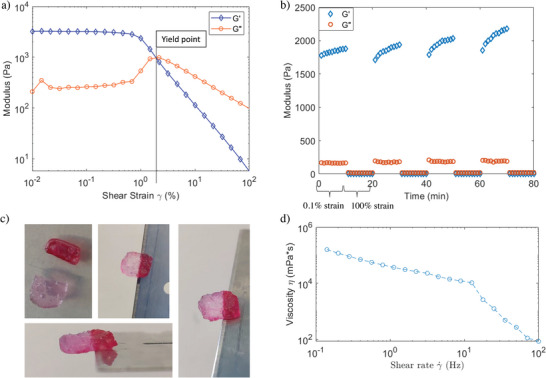
Self‐healing behavior and thixotropy of the dA/CA hydrogels. a) Modulus as a response to shear for dA_30_‐dsDNA_13_ at 700 µm. Around 1% shear strain, the *G*’’ overtakes *G*’, indicating the yield point of the gel. b) Modulus in either a low shear regime (0.1%) or a high shear (100%) reversibly breaking and reforming the material. The rapid return to initial modulus indicates self‐healing under 1 min. c) A dA_30_C_8_ 700 µm CA 20 mm CAC2NH_2_ 30 mm cut in two and stained either with GelRed or loading dye purple (up left panel) is joined back together and hung vertically (middle up image) or horizontally (bottom and right) with half of it dangling of a razor blade to demonstrate self‐healing. d) Thixotropy of hydrogel dA_30_C_4_ 700 µm, CA 20 mm.

We found that the hydrogel exhibits apparent self‐healing abilities, with complete return to its initial modulus near instantly (<1 min) following a shear induced breakage. This can be done repeatedly for at least 4 cycles without any indication of weakening. Moreover, gels cut with a razor blade exhibited clear healing behavior when the cut surfaces were brought back together (**Figure** **3**c). The self‐healing ability likely derives from the supramolecular nature of the network, in which the reversibility of bonds holding the DNA nanofibers together allow them to break and reform without lasting damage.^[^ ^21^, ^24^ ^]^ The slight increase in modulus after each cycle is likely caused by drying over the course of the 1 h experiment.

In addition to exhibiting self‐healing behavior, these hydrogels exhibited thixotropic properties—the ability to turn more fluid above a certain rate of shearing—above a shear rate of 10 Hz (Figure 3d; and Figure S21, Supporting Information). Thixotropy allows these hydrogels to be injected as easily as a liquid, but in its destination act as a solid, an important property for 3D printing and therapeutic delivery.

### 2.3 Stimuli‐Responsiveness

**Figure 4:**
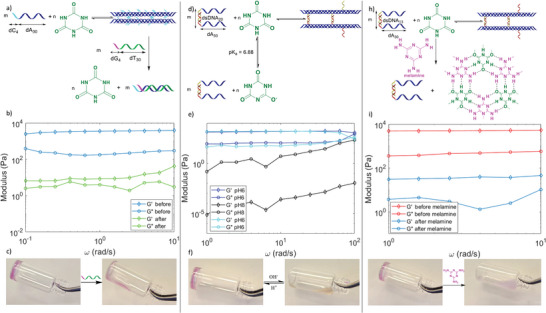
Multistimuli responsiveness of dA/CA hydrogels. Strand displacement stimuli a) reaction b) rheology (dA_30_C_4_ 700 µm, CA 20 mm) and c) final state. pH stimuli d) reaction, e) rheology (dA_30_‐dsDNA_20_ 700 µm, CA 20 mm), and f) basic state. Melamine stimuli h) reaction, i) rheology (dA_30_‐dsDNA_13_ 700 µm, CA 20 mm) and j) final state. c,f,j) are stained with Gel loading dye Purple.

A smart material is defined as being able to sense and actuate functions, requiring stimuli‐responsiveness as an essential quality.^[^ ^25^ ^]^ As the fibers making the dA/CA hydrogels are assembled in a supramolecular manner, they can revert to the free components CA and DNA in a dynamic equilibrium (**Figure** **4**). By tuning the availability of these components, the formation of fibers can be favored or disfavored, thus assembling or disassembling the hydrogel in response to stimuli.

#### 2.3.1 Strand Displacement

By adding a preferred binding partner to the DNA strand constituting the hydrogel, such as the fully complementary DNA, the equilibrium is shifted away from the fibers. In a hydrogel containing dA_30_C_4_, the addition of the complementary strand dG_4_T_30_ leads to a large loss of *G*’ and *G*″ (Figure 4a–c). This competition by an invading strand binding stronger than the dA/CA motif is similar to strand displacement,^[^ ^26^ ^]^ only the strand now displaces small molecules. This variation on the concept could be used to release a relatively large number of small molecules, some perhaps bearing therapeutic cargo, with a strand trigger. It is also an example on how the material conserves the valuable characteristics of DNA in this new hybrid assembly, while adding new characteristics based on the small molecule.

#### 2.3.2 Small‐Molecule Displacement

A competing binder can also be added to the small molecule component of the gel. A preferred binding partner of cyanuric acid is melamine,^[^ ^27^ ^]^ a molecule which allows maximum hydrogen bond formation with CA to form a hexameric rosette sheet‐like crystalline structure. Consequently, adding stoichiometric amount of melamine to a dA_30_‐dsDNA_13_ hydrogel greatly decreases *G*’ and *G*″ (Figure 4i). As melamine rips CA away from adenine in extended 2D hydrogen bonded networks, a solid precipitates from solution as white flakes, leaving behind a liquid containing the DNA strands (Figure 4j). This mirrors the previous strand‐displacement experiment, where now a small molecule trigger can release DNA strands into solution, where they could perform therapeutic,^[^ ^28^ ^]^ structural,^[^ ^7^, ^29^ ^]^ or computational tasks.^[^ ^30^ ^]^

#### 2.3.3 pH Changes

In addition to displacement by melamine, the inherent slight acidity of CA (p*K*a = 6.9) makes the hydrogels pH‐responsive. Since only the protonated form of CA can form the hydrogen bonds necessary for fiber formation, the hydrogel dissolves in basic pH (Figure 4g–i), dissociating both the strands and the CA. pH changes are readily controllable and reversible, and when the solution is again lowered to pH 6, the mechanical properties are nearly entirely recovered in only 2 min. Since the pH responsiveness happens around biological pH, it is possible to use the hydrogel as a delivery agent, unforming and releasing its cargo when added to biological samples. We will apply this property in the next sections.

### 2.4 Encapsulation, Integration, and Release

One of the main applications of hydrogels lies in cargo encapsulation and release. In this case, cargo can either be physically entrapped in the gel, or it can be integrated into the gel itself, by conjugating it to a poly‐dA strand.

#### 2.4.1 DNA Origami and Wireframe Structures

**Figure 5:**
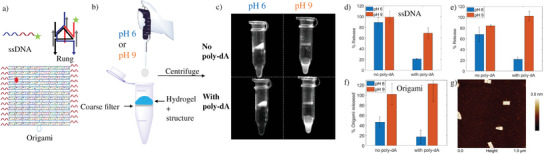
Encapsulation, integration and release of DNA nanostructures within the dA/CA hydrogels. a) Representation of the DNA nanostructures tested. ssDNA has a 1 dA_30_ region, the rung has 6 (3 on each side) while the origami has 24 (12 on each side) b) Addition of pH 6 or pH 9 buffers to dA_30_C_4_ hydrogels having the DNA nanostructures either integrated or encapsulated. The hydrogels are placed on a coarse 0.2 µm filter which allows the nanostructures to pass through, but not the dA/CA fibers. c) Cy5 fluorescent imaging of DNA origami either with or without appended dA_30_ sequences. After centrifugation, the hydrogel remains on the filter while the buffer flushes the pores and releases any encapsulated nanostructure. When the buffer is basic, the whole hydrogel is dissolved, and all its contents pass through the filter. Difference between the leakage and triggered release for d) single‐stranded DNA, e) a DNA wireframe structure (tripodal rung of a DNA nanotube) and f) DNA origami. g) AFM of the DNA origami with dA_30_ after pH triggered release showing the structure intact.

One of the advantages of using DNA hydrogels is that they allow the seamless integration of DNA nanostructures for therapeutic or diagnostic applications. While simple DNA motifs have been previously used in hydrogels, full DNA nanostructures have not been incorporated as integral parts of DNA hydrogels. We constructed a DNA triangular wireframe assembly, as well as a DNA origami rectangle, with and without extra dA_30_ polyadenine overhangs (**Figure** **5**a). These preassembled structures were added to dA_30_C_4_ hydrogels 1 min after they were cooled down to room temperature, to allow their incorporation or encapsulation during the polyadenine/CA assembly without denaturing their own structures.

To facilitate tracking of the DNA nanostructures, they each contained a strand modified with a cyanine‐3 dye. We placed the hydrogel encapsulating DNA nanostructures over a filter in a centrifuge tube, and first washed the structure with acidic buffer with centrifugation (Figure 5b). Using this eluent, almost all the DNA strand and DNA wireframe structures without poly‐dA overhangs were collected, while a lesser amount of the larger DNA origami was released, consistent with their physical encapsulation within the gel. On the other hand, only a small fraction of the DNA nanostructures with poly‐dA overhangs was collected, showing that these structures were stably integrated within the gel. By increasing the pH of the eluting buffer, the DNA nanostructures with or without poly‐dA overhangs were collected in their entirety, consistent with disintegration of the hydrogel structure at higher pH and release of the encapsulated cargo (Figure 5c,d,e,f). AFM imaging of the eluent showed that the DNA origami structures maintained their integrity after release from the hydrogel (Figure 5g). Thus, DNA nanostructures can be stably encapsulated within the DNA hydrogel at slightly acidic pH, and they can be released from the gel in a pH‐responsive manner. DNA nanostructures are already used in detection,^[^ ^31^ ^]^ computation,^[^ ^32^ ^]^ reaction control,^[^ ^33^ ^]^ as well as proposed components of future therapeutics.^[^ ^34^ ^]^ Controlling their encapsulation and release kinetics through simple sequence modification will be valuable for spatiotemporally tuning their availability in different environments.

#### 2.4.2 Release of Antisense Oligonucleotide and Gene Silencing In Vitro

**Figure 6:**
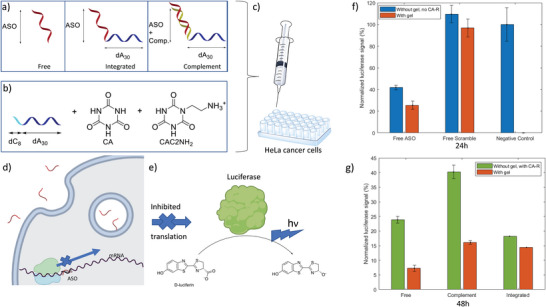
Release of antisense oligonucleotide (ASO) targeting the luciferase protein from assembled hydrogels in presence of HeLa cancer cells. a) Different methods by which the ASO (or the scramble sequence) are incorporated in the hydrogel; free, where the ASO is physically encapsulated, integrated, where the ASO has a dA30 overhang that inserts it in the gel fibers, and complement, where the ASO is hybridized to a complementary overhang sequence on the hydrogel b) Composition of the hydrogel, with the component strands dA30C8 (700 µm), CA (20 mm), and CAC2NH_2_ (30 mm) assembled in a pH buffer controlled by MES. c) The hydrogels are loaded in a syringe and injected onto plated HeLa cells. d) Scheme of one of the mechanisms of action of ASO, created with BioRender.com. e) Luciferase protein and its catalyzed reaction, used as the reporter gene in this experiment. f) 24 h time point comparing the normalized luciferase intensity with and without the gel, and compared to controls. The without gel has no CA‐R. i) 48 h timepoint comparison of the normalized luciferase signal from different incorporation methods. The without gel columns have CA‐R.

Having shown the controlled pH release of a variety of potential nucleic acids, we then applied our hydrogels to test in vitro delivery of a nucleic acid therapeutic cargo. We chose an antisense oligonucleotide (ASO), capable of silencing the reporter luciferase gene in a HeLa (cervical cancer) cell line.^[^ ^35^ ^]^ Antisense oligonucleotides are a versatile class of therapeutics, which are applied to treat diseases such as Duchenne muscular dystrophy and spinal muscular atrophy,^[^ ^36^ ^]^ with a number of other diseases currently investigated in 29 active or recruiting clinical trials as of 2022.^[^ ^37^ ^]^ They benefit from modified bases such as the 2’‐fluoroarabinose (FANA) modification, which both improves nuclease stability, binding to the target mRNA and recruitment of RNase H for degradation, resulting in high gene silencing ability.^[^ ^38^ ^]^ Our DNA hydrogels may be able to assist in the local delivery of nucleic acid therapeutics such as ASO to tissues by establishing a high local concentration, while reducing the problems associated with therapeutic levels of strands administered systemically.^[^ ^39^ ^]^ To load the FANA‐DNA (ASO) in our gel, we used three different methods, i) encapsulation of the free ASO strand (Free) in an unmodified dA/CA hydrogel, ii) hybridization of the ASO to a dA/CA hydrogel equipped with overhangs complementary to the ASO sequence (Complement), and iii) integration of the ASO into the dA/CA fibers of the hydrogel by conjugation of a dA_30_ tail to this ASO strand (Integrated) (**Figure** **6**a; and Table S4, Supporting Information). The gel itself was composed of dA_30_C_8_ strands at 700 µm, CA at 20 mm, and CAC2NH_2_ at 30 mm. We choose this composition for its temperature‐independent modulus, ensuring the release would be through pH and not heating (Figure S9, Supporting Information). The hydrogel was loaded into syringes and injected onto a plate of HeLa cells in Dulbecco's Modified Eagle Medium supplemented with 10% Fetal Bovine Serum (DMEM) buffer. Over time, its dissolution in presence of the cell environment pH released the FANA‐DNA ASO sequences. No additional transfection agents are used in this assay to decipher effects stemming from the gel itself, so a final concentration of silencing strand of 5 µm is used for gymnosis‐based entry. A scramble sequence of the ASO was used as a control, while the efficiency of silencing was compared to the same strands added only with buffer either without or with CA‐R (Figure 6f,i respectively). Silencing ability is measured by luminescence caused by the luciferase enzyme and is normalized to the number of live cells with a cell titer blue assay.

Silencing efficiency of the strands after 48 h varied between 84 ± 1% and 93 ± 1% depending on the method of incorporation, with the free strand physically encapsulated in the gel having the highest activity, and the complementary strand the lowest, possibly because of the competition in binding between the complement to the ASO and the mRNA (Figure 6i). While our goal was to obtain a similar silencing efficiency as the free strands in buffer, only locally released, we were surprised to find a stronger silencing effect when the strands were encapsulated in the hydrogels (Figure 6f). Results were normalized to cell viability, with the ASO loaded hydrogels showing similar levels of cell growth to the nonloaded hydrogels, which was in turn lower than cells grown in media (Figure S8, Supporting Information). We verified that this is not an effect of the cyanuric acid molecules added, as ASO strands in presence of these molecules, without the gel, are less effective at gene silencing (Figure 6i). We hypothesize that increased local concentration around the gel, interactions of the gel with cell membranes, and/or increased endosomal escape may explain the improved silencing ability of our hydrogel/ASO system.

## 3 Conclusion

In this work, we have developed an injectable DNA‐based hydrogel able to act as a local delivery vehicle for oligonucleotides, and to encapsulate and release DNA origami and wireframe nanostructures, selectively triggered by physiologically relevant pH. Key to this strategy is the use of a newly discovered DNA motif, where poly‐dA strands can be assembled into supramolecular fibers with the small nontoxic molecule, cyanuric acid (CA). The hydrogel can be formed by crosslinking these fibers using canonical or noncanonical DNA base‐paring: this poly‐dA/CA gel is unique in that it contains a high density of integrated small molecules along its backbone. Using this strategy, we made hydrogels out of unmodified DNA with a range of moduli from 10^1^ to 10^5^ Pa, making them the stiffest unmodified DNA hydrogels by two orders of magnitude. This large mechanical space can be navigated not only by tuning the DNA sequence but also by controlling the modifications of the small molecule cyanuric acid binding partner. The presence of the cyanuric acid imbues the material with inherent stimuli‐responsiveness to specific DNA sequences, pH and small molecules such as melamine. The supramolecular nature of the interaction allows for efficient, fast self‐healing, and thixotropy, which make it easy to use for injections of therapeutics. The hydrogel still retains the attractive advantages of DNA, with its stimuli responsiveness to strand displacement reactions and its easy integration of DNA nanostructures. DNA wireframe assemblies and DNA origami can be incorporated within the hydrogel either via physical encapsulation, or through attachment of polyadenine overhangs to these DNA nanostructures and integration into the poly‐dA/CA fibers. This ease of integration allowed us to incorporate a therapeutic nucleic acid—an antisense oligonucleotide (ASO) for luciferase—into the structure, and to release it in the presence of the physiological pH of cells. We found that, surprisingly, the presence of the hydrogel itself improved the silencing efficiency by a factor of 2–3 compared to the ASO in buffer, reaching a 95 ± 1% of silencing after 48 h under optimized conditions.

The combination of thixotropy, tunable stiffness, self‐healing behavior, stimuli‐responsiveness to pH and enhanced silencing observed in presence of this gel make it a promising material for future development of a local nucleic acid release system. The ability of CA to be functionalized with an amino group in the hydrogel could also allow attachment of drugs held in the hydrogel for multidrug stimuli‐responsive therapy.

## 4 Experimental Section

### Hydrogel Assembly and Rheology

Unless otherwise stated, the preparation of gels follows this procedure. The appropriate amounts of strands (**Table** **1**) are evaporated from their stocks on a speed vacuum in a 300 µL Eppendorf. For the dA_30_‐dsDNA*_n_* hydrogels, the concentration is half strand1 and half strand2. Once dry, 50 or 100 µL of a buffer containing the desired concentration of CA and/or CAC2NH_2_ is added to the Eppendorf. Two types of buffers were used in this work: the Mag buffer (MgCl_2_ 45.6 mm, Tris 240 mm, adjusted to pH 6 after CA addition by glacial acetic acid) and the Serum buffer (NaCl 140 mm, KCl 5 mm, Ca(NO_3_)_2_ 2.5 mm, MgCl_2_ 1.5 mm, MES 10 mm, adjust to pH 6 after CA addition with HCl). Both buffers give similar gels rheologically (Figure S4, Supporting Information). For concentrations of CA exceeding 20 mm or CAC2NH_2_ 30 mm, the excess CA‐R is dried with the strands from a water/CA 20 mm solution adjusted to pH 6. The dried DNA is mixed and sonicated to help it dissolve, forming instantly a cloudy filamentous gel. To homogenize the gels, the Eppendorf is heated to 85 °C for 5 min a Bio‐Rad T100TM thermocycler, then cooled down to 20 °C and kept at that temperature overnight, unless otherwise noted. They are measured the next day. It is important to properly homogenize the gels, mixing them at high temp if needed, and to quickly cool them, or they may form more localized aggregates with varying rheology. Due to the high melting temperature of dA_30_C_8_ 2 mm, CA 60 mm, CAC2NH_2_ 90 mm, the gel was prepared by heating at 100 °C for 20 min (sealed to prevent water escaping), before being brought to 20 °C. Clear monophasic gels are formed from this process (Table 1).

**Table 1:** Sequences of DNA strands used for hydrogels

Name	Sequence (5’ → 3’)
dA_15_	AAAAAAAAAAAAAAA
dA_30_	AAAAAAAAAAAAAAAAAAAAAAAAAAAAAA
dA_50_	AAAAAAAAAAAAAAAAAAAAAAAAAAAAAAAAAAAAAAAAAAAAAAAAAA
dA_30_C_4_	AAAAAAAAAAAAAAAAAAAAAAAAAAAAAACCCC
dA_30_C_8_	AAAAAAAAAAAAAAAAAAAAAAAAAAAAAACCCCCCCC
dA_30_G_4_	AAAAAAAAAAAAAAAAAAAAAAAAAAAAAAGGGG
dA_30_G_8_	AAAAAAAAAAAAAAAAAAAAAAAAAAAAAAGGGGGGGG
dA_30_‐dsDNA_13_‐strand1	AAAAAAAAAAAAAAAAAAAAAAAAAAAAAACTAGAAGTGTCCAGTTA
dA_30_‐dsDNA_13_‐strand2	AAAAAAAAAAAAAAAAAAAAAAAAAAAAAATTACTAACTGGACACTT
dA_30_‐dsDNA_20_‐strand1	AAAAAAAAAAAAAAAAAAAAAAAAAAAAAACCCCATTCTACTTGAGAGAGCGAC
dA_30_‐dsDNA_20_‐strand2	AAAAAAAAAAAAAAAAAAAAAAAAAAAAAACCCCGTCGCTCTCTCAAGTAGAAT

### Rheological Measurements

Rheological properties were measured using stress‐controlled rheometer (MCR 302, Anton Paar) using a 15 mm diameter cone plate at 0.2 mm (50 µL gels) or 0.5 mm (100 µL gels) gap distance. Samples were taken directly from the Eppendorf in which they were formed by prying it open with a razor blade and placed under the cone plate. The temperature was maintained at 25 °C for all measurements using a Peltier temperature control unit unless otherwise noted. Due to the small thickness of the samples, no temperature gradients are expected. To avoid drying, a solvent trap was used in all the measurements. Amplitude sweeps were first conducted to assess the linear viscoelastic range of the samples by slowly increasing the strain from 0.01% to 100%. The yield point was found to be around 1%. Oscillatory frequency sweep tests were measured between 0.1 and 100 Hz at 0.1% strain. Strain‐cycle experiment was done by cycling the gel between 0.1% and 100% strains at 1 Hz. To understand the shear‐thinning behavior of the samples, the viscosity was measured at increasing strain rate ranging from (0.1 to 100 Hz).

### Stimuli Responsiveness

a) Strand complement

Two 50 µL dA_30_C_4_ 700 µm hydrogel in Mag buffer with CA 20 mm samples were prepared, as well as a 10 µL sample of dG_4_T_30_ 3 mm (1.5 eq.) in the same buffer and CA concentration. A sample of dA_30_C_4_ hydrogel was measured independently. The dG_4_T_30_ sample was added to the second dA_30_C_4_ sample and vortexed in its Eppendorf for 2 min before being measured.

b) Melamine

A 50 µL hydrogel composed of dA_30_‐dsDNA_13_ 700 µm and CA 20 mm in Mag buffer was measured in the rheometer. The plate of the rheometer was lifted and 50 µL of solution of 20 mm melamine in Mag buffer at pH6 was added to it. Instant white precipitate formed and was encouraged by gently mixing with a spatula for 30 s before the resulting liquid with solid precipitate was measured rheologically.

c) pH

A volume of 180 µL of dA_30_‐dsDNA_20_ 700 µm CA 20 mm hydrogel in Mag buffer was prepared. The initial pH was measured at 5.71 with a surface pH meter (Mettler Toledo). An approximate volume of 50 µL was separated and measured rheologically. To the reminding 130 µL, 2.70 µL of NaOH 5.04 m was added and stirred through vortexing. The resulting pH of the liquid was measured at 7.91. 50 µL of it were measured rheologically. To the remaining 80 µL, 1.5 µL of HCl 5.04 m was added by aliquot and mixed through vortexing, forming a gel with a pH of 5.66 after 2 min. To note that in general, it is important to mix vigorously with the addition of the acid, or localized gels will form rather than a homogeneous one. That gel was then analyzed rheologically.

### SEM and AFM Imaging

A 100 µL dA_30_‐dsDNA_13_ 700 µm in CA 20 mm Mag buffer was prepared. It was frozen with liquid nitrogen then dried in freeze‐drier overnight. The dried sample was placed on carbon tape and imaged by Hitachi SU3500 scanning electron microscope equipped with ultravariable pressure detector operating at 10 kV accelerating voltage under 30 Pa pressure.

A hydrogel with the same composition was broken down by adding 3 mL of Mag buffer and strongly mixing with the pipette, then 5 µL of the sample was dropped onto a freshly cleaved mica surface for 30 s, followed by wicking off most of the liquid from the mica surface using a filter paper. The mica surface was then further dried under a stream of compressed air for 30 s before it was put under vacuum for at least 2 h prior to imaging. AFM images were acquired in ScanAsyst mode under air conditions on a Multimode 8 Scanning Probe Microscope from Bruker with a Nanoscope V controller equipped with a ScanAsyst‐Air silicon tip on nitride lever (tip radius = 2 nm, *k* = 0.4 N m^−1^, *f*_o_ = 70 kHz; Bruker).

### Nanostructure Assembly, Encapsulation, Integration, and Release

The design and assembly of the rectangle DNA origami structure functionalized with poly‐dA strands were based on the method reported by Rothemund et al.,^[^ ^40^ ^]^ while the wireframe rung was based on the method reported by Rahbani et al.^[^ ^41^ ^]^ For the origami, the long circular single‐stranded viral scaffold M13mp18 (purchased from Guild BioSciences, USA) was folded into a rectangular tile with the aids of short single‐stranded staple strands (Tables S2 and S3, Figures S6 and S7 (Supporting Information), purchased from Bioneer, Inc., USA). A single staple strand was synthesized on a Mermade MM6 synthesizer with a 3’‐Cy5 dye. The origami structure was assembled in one‐pot, where 3 nm M13mp18 scaffold and 45 nm each staple strands were mixed and heated to and held at 95 °C for 5 min in 1×TAMg buffer (pH = 8.3) and slowly annealed to 20 °C (−1 °C min^−1^). To remove excess staple strands and adapt the solution pH to 6.7 (compatible with dA/CA fiber formation), the annealed sample was washed in a 100 kDa Amicon centrifugal filter Unit. First, 500 µL samples were centrifuged at 6500 rpm at 4 °C for 5 min. Then, 400 µL 1×TAMg (pH = 6.7) was added and the samples were centrifuged again at 5000 rpm at 4 °C for 6 min. This filtration step was repeated four more times. Approximately 50–100 µL samples were recovered after the purification and were quantified by a NanoDrop Lite Spectrophotometer (ThermoFisher Scientific).

For the wireframe rung, it is formed by mixing all component strands either with or without poly‐dA_30_ overhangs (Table S1, Supporting Information) in equimolar concentrations to a final concentration of 0.30 µm in 1×TAMg. A single strand per construct contained a 3’‐Cy3 dye. The mixture was annealed from 95 to 20 °C over 6 h resulting in a quantitative yield of the rung.

A 100 µm hydrogel of dA_30_C_4_ 500 µm (CA 20 mm, Mag buffer) was brought to 85 °C for 5 min, and cooled to 20 °C for 1 min, then the constructs are added for a final concentration of 1 nm (Origami) or 40 nm (rung and ssDNA) and left to cool at 20 °C for 15 min. The gels are then placed on a filter of a Freeze 'N Squeeze DNA Gel Extraction Spin Columns (BioRad). Either 100 µL of Mag buffer at pH 6 or pH 9 is added to them for 25 min, then the column are spin down at 6k for 6 min. The filtrate is collected, mixed gently, and analyzed by fluorescence of the Cy3 or Cy5 dye in triplicate on a Biotek Synergy HT plate reader. The concentration is obtained through a calibration curve. An origami filtrate from the pH 9 was collected and 5 µL of it was dropped on freshly cleaved mica and imaged on AFM.

### In Vitro Silencing and Viability Assays

Hydrogels were formed using the sequences in Table S4 (Supporting Information). The ASO, Scramble, ASO‐integrated, and Scramble integrated are synthesized using a Mermade MM6 synthesizer from Bioautomation, then purified using gel‐electrophoresis. The ASO‐Complement, Scramble‐Complement, and dA_30_C_8_ were all purchased from Integrated DNA Technologies desalted and used as is. **FANA**‐DNA strands were synthesized in house as described elsewhere.^[^ ^42^ ^]^

Hydrogels were prepared by using a CA 20 mm, CAC2NH_2_ 30 mm Serum buffer adjusted to pH 6, sterilized by filtration through a 0.2 µm Nylon Centrifugal Filters from Canadian Life Sciences. The gelling strands of dA_30_C_8_ were at 700 µm, the therapeutic strands (ASO, Scramble, ASO‐integrated or Scramble‐Integrated) at 15 µm, and, for the complement samples, the complement strands (ASO‐complement, Scramble‐Complement) at 30 µm in a total volume of 100 µL. They were heated to 85 °C for 20 min before being aspirated into 1 mL syringes while hot. The syringes were left at room temperature for 1 h before being used.

Luciferase expressing HeLa cells were maintained in DMEM containing 10% FBS supplemented with antibiotic/antimycotics at 37 °C, 5% CO_2_. Cells were passed every 3 days in a ratio of 1:5. Luciferase knockdown assays were performed by plating 50 000 cells per well, for 24 h experiments, or 25 000 cells per well, for 48 h experiments, in a 24 well plate. Cells were incubated overnight at 37 °C, 5% CO_2_ to allow for adhesion to the plate. Following incubation, 100 µL of either hydrogel or buffer solutions were added in duplicate (48 h) or quadruplicate (24 h) to wells directly, as well as 200 µL of fresh DMEM with 10% FBS. Cells were then incubated for either 24 or 48 h postaddition of samples.

Cytotoxicity and cell viability was analyzed by incubating cells with using 100 µL of a premade fluorescent reagent (Celltiter Blue) for 2 h at 37 °C, 5% CO_2_. Fluorescence from the 24 well plates was then measured Ex. 530 nm, Em. 590 nm using Biotek Synergy HT, using BioTek Gen 4 Software (Figure S8, Supporting Information).

Luciferase assay was performed by first removing media from cells then adding 200 µL of a 1:1 Mix of Promega Glo‐Lysis Buffer and Promega Bright‐Glo Luciferase Assay System to each well. Luminescence was measured at 528 nm using Biotek Synergy HT, using BioTek Gen 4 Software. Luciferase silencing was corrected with cell viability and normalized to cells with a simple 1xPBS addition.

## Conflict of Interest

The authors declare no conflict of interest.

## Supporting information

Supporting Information

## Data Availability

The data that support the findings of this study are available from the corresponding author upon reasonable request.

## References

[1] a) O. Jeon , K. Lee , E. Alsberg , Small 2018, 14, e1800579.; 29782703 10.1002/smll.201800579 PMC6238642

[2] a) X. Huang , R. Zheng , F. Ding , J. Yang , M. Xie , X. Liu , J. Li , J. Feng , X. Zhu , C. Zhang , ACS Mater. Lett. 2020, 2, 1509;

[3] Y. Shao , Z.‐Y. Sun , Y. Wang , B.‐D. Zhang , D. Liu , Y.‐M. Li , ACS Appl. Mater. Interfaces 2018, 10, 9310. 29484882 10.1021/acsami.8b00312

[4] S. Kim , J. Fan , C.‐S. Lee , C. Chen , M. Lee , ACS Appl. Bio. Mater. 2021, 4, 5189. 10.1021/acsabm.1c00369 PMC8513506 34661086

[5] a) F. Huang , M. Chen , Z. Zhou , R. Duan , F. Xia , I. Willner , Nat. Commun. 2021, 12, 2364; 33888708 10.1038/s41467-021-22645-8 PMC8062675

[6] Y. Gu , M. E. Distler , H. F. Cheng , C. Huang , C. A. Mirkin , J. Am. Chem. Soc. 2021, 143, 17200. 34614359 10.1021/jacs.1c08114

[7] a) M. Oishi , K. Nakatani , Small 2019, 15, e1900490. 30859712 10.1002/smll.201900490

[8] L. Yue , S. Wang , Z. Zhou , I. Willner , J. Am. Chem. Soc. 2020, 142, 21577. 33325692 10.1021/jacs.0c09891

[9] R. Merindol , G. Delechiave , L. Heinen , L. H. Catalani , A. Walther , Nat. Commun. 2019, 10, 528. 30705271 10.1038/s41467-019-08428-2 PMC6355893

[10] J. Shi , C. Zhu , Q. Li , Y. Li , L. Chen , B. Yang , J.‐F. Xu , Y. Dong , C. Mao , D. Liu , Macromol. Rapid Commun. 2021, 42, 2100182. 10.1002/marc.202100182 34028914

[11] C. F. Guimarães , L. Gasperini , A. P. Marques , R. L. Reis , Nat. Rev. Mater. 2020, 5, 351.

[12] M. Madsen , K. V. Gothelf , Chem. Rev. 2019, 119, 6384. 30714731 10.1021/acs.chemrev.8b00570

[13] N. Avakyan , A. A. Greschner , F. Aldaye , C. J. Serpell , V. Toader , A. Petitjean , H. F. Sleiman , Nat. Chem. 2016, 8, 368. 27001733 10.1038/nchem.2451

[14] a) C. Lachance‐Brais , C. D. Hennecker , A. Alenaizan , X. Luo , V. Toader , M. Taing , C. D. Sherrill , A. K. Mittermaier , H. F. Sleiman , J. Am. Chem. Soc. 2021, 143, 19824; 34783562 10.1021/jacs.1c08972

[15] F. J. Rizzuto , C. M. Platnich , X. Luo , Y. Shen , M. D. Dore , C. Lachance‐Brais , A. Guarné , G. Cosa , H. F. Sleiman , Nat. Chem. 2021, 13, 843. 34373598 10.1038/s41557-021-00751-w

[16] D. Calvet , J. Y. Wong , S. Giasson , Macromolecules 2004, 37, 7762.

[17] a) W. Helen , P. de Leonardis , R. V. Ulijn , J. Gough , N. Tirelli , Soft Matter 2011, 7, 1732;

[18] a) Z. Yang , B. Xu , Chem. Commun. 2004, 2424; 10.1039/b408897b 15514797

[19] a) Q. Hu , K. Dong , J. Ming , W. Yang , H. Wang , X. Xiao , T. Huang , Mater. Today Chem. 2022, 23, 100680;

[20] a) S. Basu , A. Chakraborty , A.‐R. I. Alkiswani , Y. Shamiya , A. Paul , Mater. Adv. 2022, 3, 946;

[21] Y. Shao , H. Jia , T. Cao , D. Liu , Acc. Chem. Res. 2017, 50, 659. 28299927 10.1021/acs.accounts.6b00524

[22] L. L. Wang , J. J. Chung , E. C. Li , S. Uman , P. Atluri , J. A. Burdick , J. Controlled Release 2018, 285, 152. 10.1016/j.jconrel.2018.07.004 PMC6134398 29981357

[23] C. B. Highley , C. B. Rodell , J. A. Burdick , Adv. Mater. 2015, 27, 5075. 26177925 10.1002/adma.201501234

[24] S. Seiffert , J. Sprakel , Chem. Soc. Rev. 2012, 41, 909. 21909565 10.1039/c1cs15191f

[25] R. E. Newnham , G. R. Ruschau , J. Am. Ceram. Soc. 1991, 74, 463.

[26] F. C. Simmel , B. Yurke , H. R. Singh , Chem. Rev. 2019, 119, 6326. 30714375 10.1021/acs.chemrev.8b00580

[27] E. E. Simanek , X. Li , I. S. Choi , G. M. Whitesides , Templating, Self‐Assembly and Self‐Organization, Elsevier, Amsterdam 1996, pp. 595–621.

[28] Y. Cai , E. Lopez‐Ruiz , J. Wengel , L. B. Creemers , K. A. Howard , J. Controlled Release 2017, 253, 153. 10.1016/j.jconrel.2017.03.004 28274742

[29] a) D. Wang , L. Yu , C.‐M. Huang , G. Arya , S. Chang , Y. Ke , J. Am. Chem. Soc. 2021, 143, 2256; 33529009 10.1021/jacs.0c10576

[30] L. Qian , E. Winfree , J. Bruck , Nature 2011, 475, 368. 21776082 10.1038/nature10262

[31] S. Wang , Z. Zhou , N. Ma , S. Yang , K. Li , C. Teng , Y. Ke , Y. Tian , Sensors 2020, 20, 6899. 33287133 10.3390/s20236899 PMC7731452

[32] a) H. Pei , L. Liang , G. Yao , J. Li , Q. Huang , C. Fan , Angew. Chem., Int. Ed. 2012, 51, 9020; 10.1002/anie.201202356 22887892

[33] a) S. Fan , B. Ji , Y. Liu , K. Zou , Z. Tian , B. Dai , D. Cui , P. Zhang , Y. Ke , J. Song , Angew. Chem., Int. Ed. 2022, 61, e202116324; 10.1002/anie.202116324 34931420

[34] S. Jiang , Z. Ge , S. Mou , H. Yan , C. Fan , Chem 2021, 7, 1156.

[35] H. H. Fakih , A. Katolik , E. Malek‐Adamian , J. J. Fakhoury , S. Kaviani , M. J. Damha , H. F. Sleiman , Chem. Sci. 2021, 12, 2993. 34164068 10.1039/d0sc06645a PMC8179377

[36] Y. Zhu , L. Zhu , X. Wang , H. Jin , Cell Death Dis. 2022, 13, 644. 35871216 10.1038/s41419-022-05075-2 PMC9308039

[37] U.S. National Library of Medicine, clinicaltrials.gov, 2022.

[38] a) A. Kalota , L. Karabon , C. R. Swider , E. Viazovkina , M. Elzagheid , M. J. Damha , A. M. Gewirtz , Nucl. Acids Res. 2006, 34, 451; 16421272 10.1093/nar/gkj455 PMC1342038

[39] N. Carballo‐Pedrares , I. Fuentes‐Boquete , S. Díaz‐Prado , A. Rey‐Rico , Pharmaceutics 2020, 12, 752. 32785171 10.3390/pharmaceutics12080752 PMC7464633

[40] P. W. K. Rothemund , Nature 2006, 440, 297. 16541064 10.1038/nature04586

[41] J. F. Rahbani , A. A. Hariri , G. Cosa , H. F. Sleiman , ACS Nano 2015, 9, 11898. 26556531 10.1021/acsnano.5b04387

[42] C.‐N. Lok , E. Viazovkina , K.‐L. Min , E. Nagy , C. J. Wilds , M. J. Damha , M. A. Parniak , Biochemistry 2002, 41, 3457. 11876654 10.1021/bi0115075

