# Supplementary material for: Small Molecule‐Templated DNA Hydrogel with Record Stiffness Integrates and Releases DNA Nanostructures and Gene Silencing Nucleic Acids
Source: Adv Sci (Weinh). 2023 Feb 8;10(12):2205713. doi: 10.1002/advs.202205713 (PMC10131789; doi:10.1002/advs.202205713)
Supplement: Supplementary file 1 — Supporting Information [file ADVS-10-2205713-s001.pdf]

ADVANCED SCIENCE
Open Access

Supporting Information

for *Adv. Sci.*, DOI 10.1002/advs.202205713

Small Molecule-Templated DNA Hydrogel with Record Stiffness Integrates and Releases DNA Nanostructures and Gene Silencing Nucleic Acids

*Christophe Lachance-Brais, Mostafa Rammal, Jathavan Asohan, Adam Katolik, Xin Luo, Daniel Saliba, Antranik Jonderian, Masad J. Damha, Matthew J. Harrington and Hanadi F. Sleiman**

Supporting Information

Small molecule-templated DNA hydrogel with record stiffness integrates and releases DNA nanostructures and gene silencing nucleic acids

*Christophe Lachance-Brais, Mostafa Rammal, Jathavan Asohan, Adam Katolik, Xin Luo, Daniel Saliba, Antranik Jonderian, Masad J. Damha, Matthew Harrington, Hanadi F. Sleiman**

Materials

The strands were purchased desalted from Integrated DNA Technologies (IDT) unless otherwised noted. Cyanuric acid (CA) 98%, 4-morpholineethanesulfonic acid hydrate (MES), $MgCl_2$*6H_2O, $Ca(NO_3)_2$*2H_2O, KCl, NaCl, tris(hydroxymethyl)aminomethane (Tris), glacial acetic acid, urea, EDTA were obtained from Sigma-Aldrich. $CAC2NH_2$ was synthesized as previously described.[14a]

Cyanine 3 (Cy3) and Cyanine 5 (Cy5) phosphoramidite were purchased from GlenResearch.

Dulbecco's Modified Eagle Medium (DMEM) and phosphate-buffered saline (PBS) were purchased from Life Technologies. Fetal bovine serum (FBS), 0.05% Trypsin-EDTA, sodium pyruvate were obtained from Wisent Bioproducts. Bright-Glo Luciferase assay and Cell-Titer Blue assay was obtained from Promega. The luciferase-expressing HeLa X1/5 cells are a generous gift from Dr. Pelletier (McGill).

Instrumentation

Circular Dichroism (CD) were performed on JASCO J-810 spectropolarimeter equipped with a Peltier temperature controller, a xenon lamp and a water recirculator with a 0.1 mm path length quartz cuvette. UV-vis measurments were performed on a Cary 300 Series UV-Vis Spectrophotometer from Agilent connected to a water recirculator with a 1 mm quartz cuvette. Quantification of DNA and RNA were performed with a Nanodrop Lite spectrophotometer from Thermo Scientific.

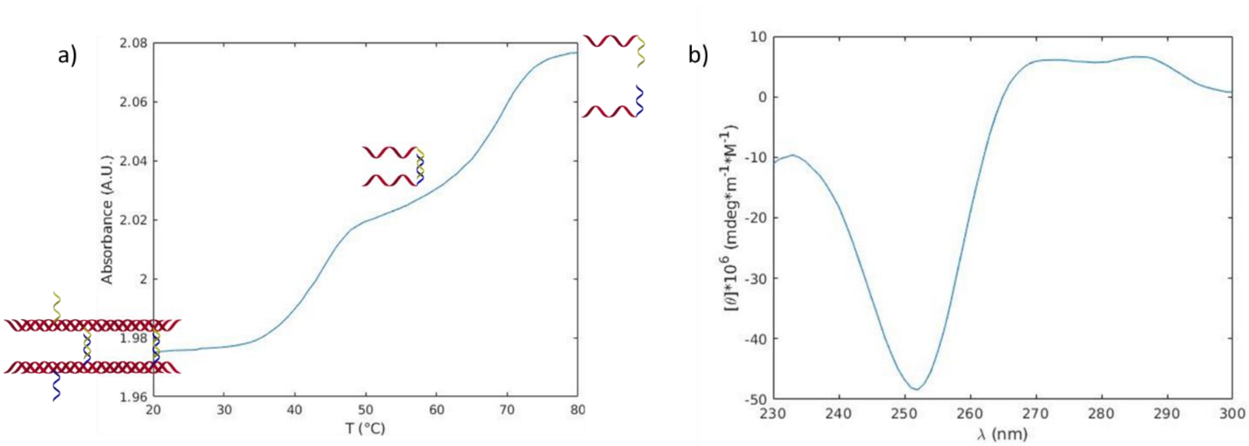

Figure S1. Demonstration of the compatibility of the dA/CA motif and double-stranded DNA by UV-vis. a) UV-vis spectra of annealing of the dA_{30}-$dsDNA_{20}$ strands (50µM, CA 20mM, Mag buffer, 80˚C to 20˚C, 2˚C/min) showing the double transition associated with its 2 constituent motifs. (double-stranded DNA at 68 ˚C , theoretical T_m = 69.5 ˚C by the IDT oligo analyzer online tool, dA/CA at 42˚C) b) Final circular dichroism spectra of the annealed strands showing the characteristic signal of the dA/CA motif, demonstrating its presence.

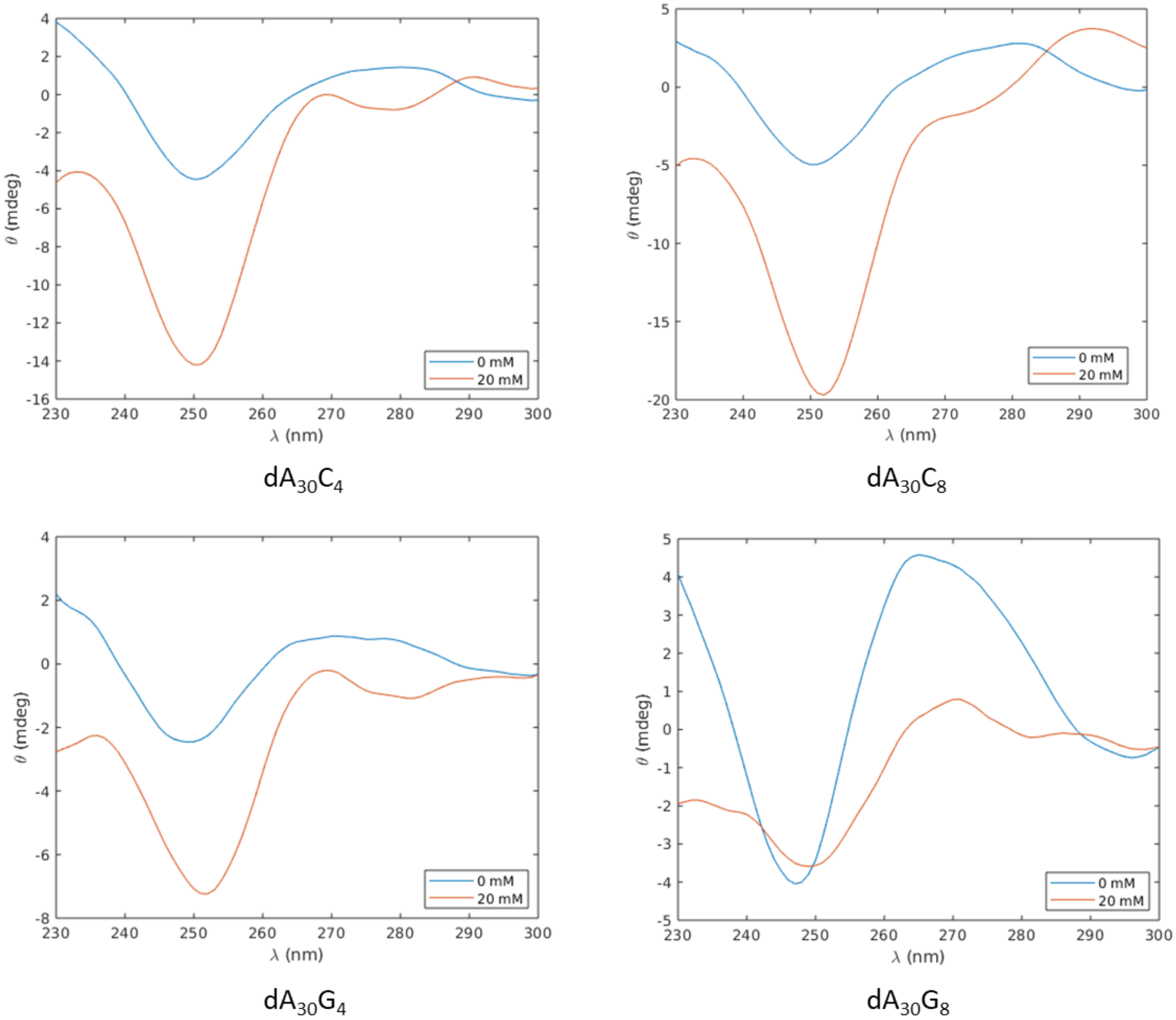

Figure S2. Circular dichroism spectra of i-motif and G-quadruplex forming strands (25µM, Mag buffer) with or without the presence of CA. a-b) The i-motif strands show the appearance of the 295 nm band associated with i-motif only when CA is present. c) For the $dA_{30}G_4$ strand, a faint increase in the 270 nm region as well as a partial cancelling of the dip in the 250-260 nm region suggest the formation of a G-quadruplex only when the CA is present. d) Without the addition of CA, the presence of a parallel G-quadruplex is clear for the $dA_{30}G_8$ with the large positive peak at 260 nm followed by a dip in the 240-250 nm region. With CA, the signal is fainter, but the presence of the shoulder at 260 nm and the shift of the dip minima from the conventional 252 nm to 249 nm suggests the superposition of a parallel G-quadruplex signal with the dA/CA signal

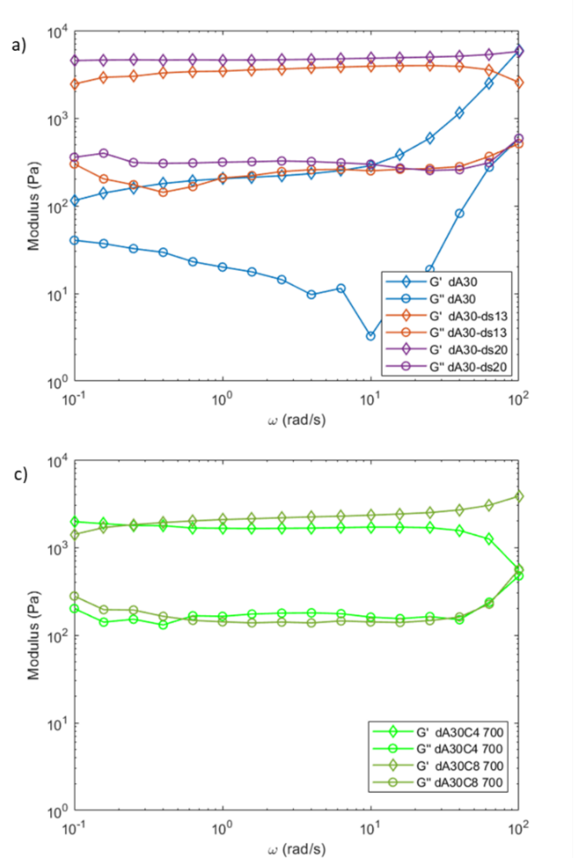

Figure S3. Comparison of the rheological properties of different crosslinking sequences and their lengths (CA 20mM, Mag buffer, 700μM strands). Comparison between a) double-stranded DNA b) G-quadruplex and c) i-motif.

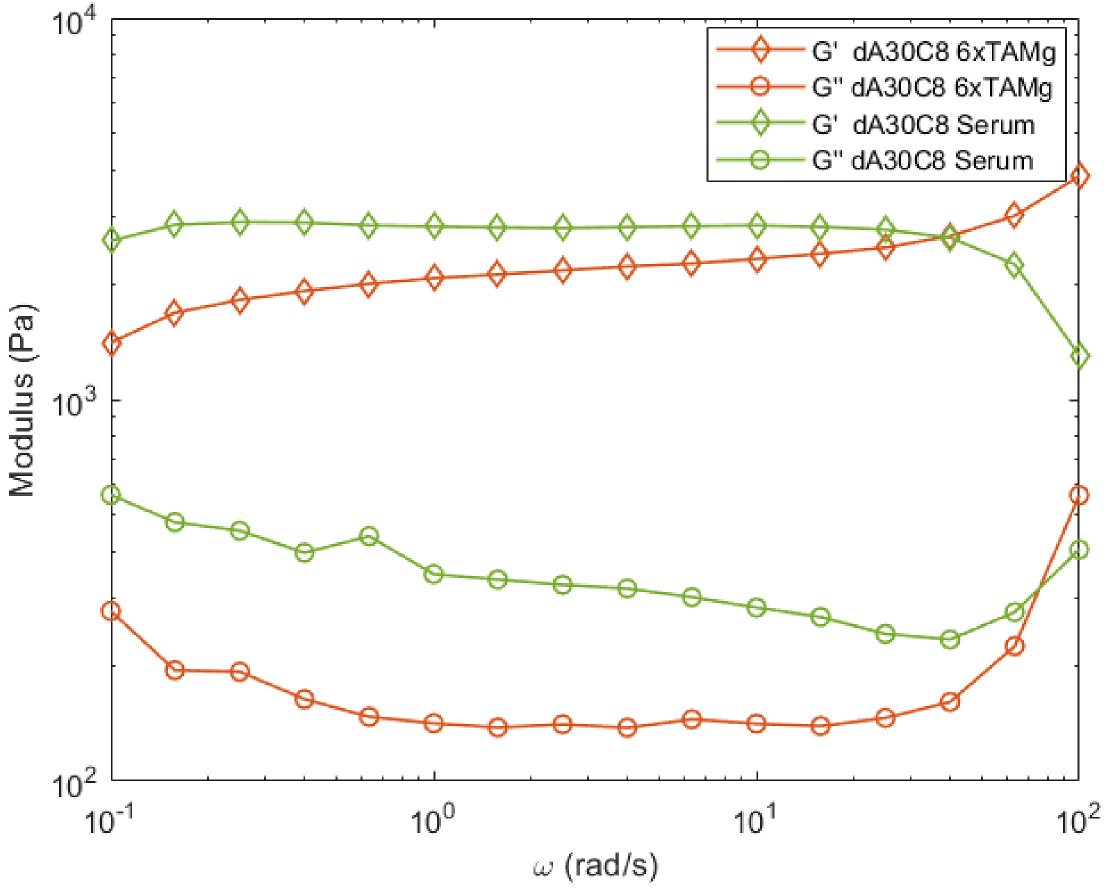

Figure S4. Rheology of $dA_{30}C_8$ 700µM and CA 20mM in the Mag and Serum buffers.

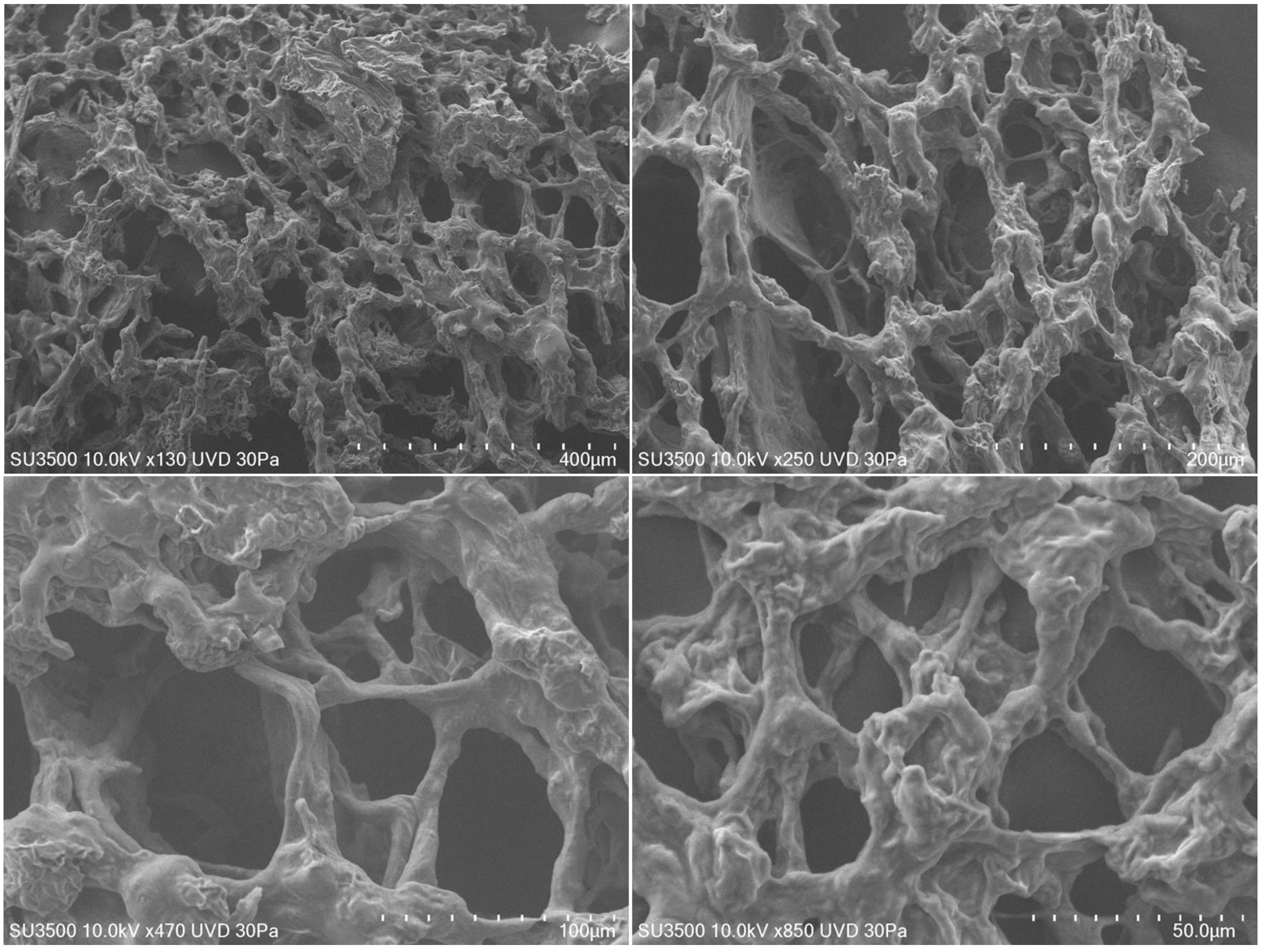

Figure S5. SEM images of dA_{30}-$dsDNA_{13}$ 700µM (CA 20mM, Mag buffer) at different length scales.

Table S1. Oligonucleotide sequences used in the assembly of wireframe rung unit. The triangular rung unit is formed by mixing all component strands (V, C1, C2, R1 cap polyA30, R2 cap polyA30, and R3 cap polyA30 or V, C1, C2, R1 cap, R2 cap, and R3 cap)

Name	Sequence (5' → 3')
**V**	CTCAGCAGCGAAAAACCGCTTTACAACATTCGAGGCACGTTGTAC GTCCACACTTGGAACCTCATCGCACATCCGCCTGCCACGCTCTTA GCATAGGACGGCGGCGTTAAATA
**C1**	CGGTGCATTTCGACGGTACTTCGTACAACGTGCCTCGAATGTAGA GCGTGGCAGGCGGATGTGAAGCAGTTGCAGCGTACTCGT
**C2**	TCGGCAGACTAATACACCTGTCGATGAGGTTCCAAGTGTGGATAG CTAGGTAACGGATTGAGC

**R1 cap poly-dA_{30}**	AAAAAAAAAAAAAAAAAAAAAAAAAAAAAACGAGTACGCTGCAACTGCTACCAGGTGTATTAGTCTGCCGAAAAAAAAAAAAAAAAAAAAAAAAAAAAAA
**R2 cap poly-dA_{30}**	AAAAAAAAAAAAAAAAAAAAAAAAAAAAAACTCAATCCGTTACCTAGCTCCAGTACCGTCGAAATGCACCAAAAAAAAAAAAAAAAAAAAAAAAAAAAAA
**R3 cap poly-dA_{30}**	AAAAAAAAAAAAAAAAAAAAAAAAAAAAAAAACGCCGCCGTCCTATGCTTTGTAAAGCGGTTTTTCGCTGAAAAAAAAAAAAAAAAAAAAAAAAAAAAAA
**R1 cap**	CGAGTACGCTGCAACTGCTACCAGGTGTATTAGTCTGCCG
**R2 cap**	CTCAATCCGTTACCTAGCTCCAGTACCGTCGAAATGCACC
**R3 cap**	CGCCGCCGTCCTATGCTTTGTAAAGCGGTTTTTCGCTG

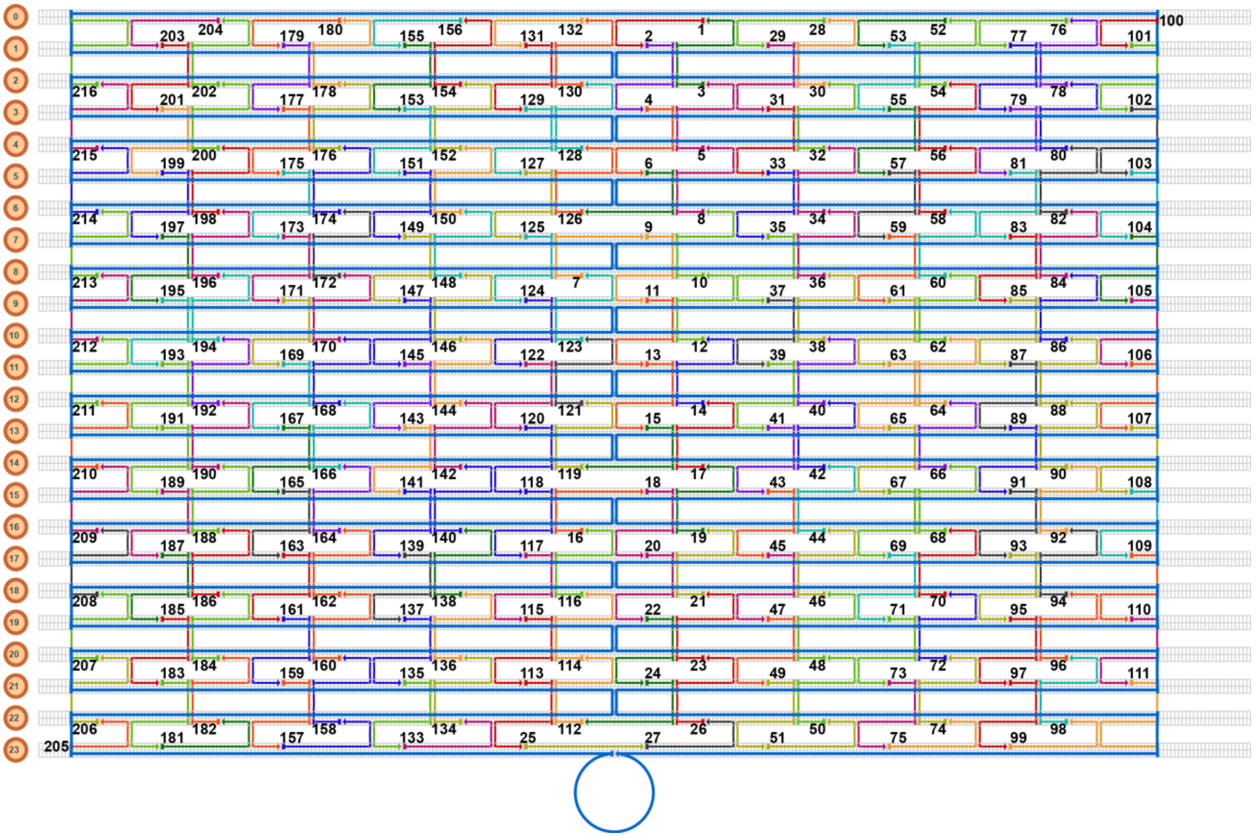

Figure S6. The detailed single-layered rectangular DNA origami design where original staple strand 1-216 are marked at their 5' end. Original staple strand sequences are listed in Table S2. Strand 175 contains a single Cy5 dye at its 3' end.

Table S2. Staple strand sequences for the origami without poly-dA.

Unmodified staple strands (1-216)

1	CAAGCCCAATAGGAACCCATGTACAAACAGTT
2	AATGCCCCGTAACAGTGCCCGTATCTCCCTCA
3	TGCCTTGACTGCCTATTTCGGAACAGGGATAG
4	GAGCCGCCCCACCACCGGAACCGCGACGGAAA
5	AACCAGAGACCCTCAGAACCGCCAGGGGTCAG
6	TTATTCATAGGGAAGGTAAATATTCATTCAGT
7	CATAACCCGAGGCATAGTAAGAGCTTTTTAAG
8	ATTGAGGGTAAAGGTGAATTATCAATCACCGG
9	AAAAGTAATATCTTACCGAAGCCCTTCCAGAG
10	GCAATAGCGCAGATAGCCGAACAATTCAACCG
11	CCTAATTTACGCTAACGAGCGTCTAATCAATA
12	TCTTACCAGCCAGTTACAAAATAAATGAAATA
13	ATCGGCTGCGAGCATGTAGAAACCTATCATAT
14	CTAATTTATCTTTCCTTATCATTCATCCTGAA
15	GCGTTATAGAAAAAGCCTGTTTAGAAGGCCGG
16	GCTCATTTTCGCATTAAATTTTTGAGCTTAGA
17	AATTACTACAAATTCTTACCAGTAATCCCATC
18	TTAAGACGTTGAAAACATAGCGATAACAGTAC
19	TAGAATCCCTGAGAAGAGTCAATAGGAATCAT
20	CTTTTACACAGATGAATATACAGTAAACAATT
21	TTTAACGTTCGGGAGAAACAATAATTTTCCCT
22	CGACAACTAAGTATTAGACTTTACAATACCGA
23	GGATTTAGCGTATTAAATCCTTTGTTTTCAGG
24	ACGAACCAAAACATCGCCATTAAATGGTGGTT
25	GAACGTGGCGAGAAAGGAAGGGAACAAACTAT
26	TAGCCCTACCAGCAGAAGATAAAAACATTTGA
27	CGGCCTTGCTGGTAATATCCAGAACGAACTGA
28	CTCAGAGCCACCACCCTCATTTTCCTATTATT
29	CTGAAACAGGTAATAAGTTTTAACCCCTCAGA
30	AGTGTACTTGAAAGTATTAAGAGGCCGCCACC
31	GCCACCACTCTTTTCATAATCAAACCGTCACC
32	GTTTGCCACCTCAGAGCCGCCACCGATACAGG
33	GACTTGAGAGACAAAAGGGCGACAAGTTACCA
34	AGCGCCAACCATTTGGGAATTAGATTATTAGC
35	GAAGGAAAATAAGAGCAAGAAACAACAGCCAT
36	GCCCAATACCGAGGAAACGCAATAGGTTTACC
37	ATTATTTAACCCAGCTACAATTTTCAAGAACG
38	TATTTTGCTCCCAATCCAAATAAGTGAGTTAA
39	GGTATTAAGAACAAGAAAAATAATTAAAGCCA
40	TAAGTCCTACCAAGTACCGCACTCTTAGTTGC
41	ACGCTCAAAATAAGAATAAACACCGTGAATTT
42	AGGCGTTACAGTAGGGCTTAATTGACAATAGA
43	ATCAAAATCGTCGCTATTAATTAACGGATTCG
44	CTGTAAATCATAGGTCTGAGAGACGATAAATA
45	CCTGATTGAAAGAAATTGCGTAGACCCGAACG
46	ACAGAAATCTTTGAATACCAAGTTCCTTGCTT
47	TTATTAATGCCGTCAATAGATAATCAGAGGTG
48	AGATTAGATTTAAAAGTTTGAGTACACGTAAA
49	AGGCGGTCATTAGTCTTTAATGCGCAATATTA
50	GAATGGCTAGTATTAACACCGCCTCAACTAAT
51	CCGCCAGCCATTGCAACAGGAAAAATATTTTT
52	CCCTCAGAACCGCCACCCTCAGAACTGAGACT
53	CCTCAAGAATACATGGCTTTTGATAGAACCAC
54	TAAGCGTCGAAGGATTAGGATTAGTACCGCCA
55	CACCAGAGTTCGGTCATAGCCCCCGCCAGCAA
56	TCGGCATTCCGCCGCCAGCATTGACGTTCCAG
57	AATCACCAAATAGAAAATTCATATATAACGGA
58	TCACAATCGTAGCACCATTACCATCGTTTTCA
59	ATACCCAAGATAACCCACAAGAATAAACGATT
60	ATCAGAGAAAGAACTGGCATGATTTTATTTTG
61	TTTTGTTTAAGCCTTAAATCAAGAATCGAGAA
62	AGGTTTTGAACGTCAAAAATGAAAGCGCTAAT
63	CAAGCAAGACGCGCCTGTTTATCAAGAATCGC
64	AATGCAGACCGTTTTTATTTTCATCTTGCGGG
65	CATATTTAGAAATACCGACCGTGTTACCTTTT
66	AATGGTTTACAACGCCAACATGTAGTTCAGCT
67	TAACCTCCATATGTGAGTGAATAAACAAAATC
68	AAATCAATGGCTTAGGTTGGGTTACTAAATTT
69	GCGCAGAGATATCAAAATTATTTGACATTATC
70	AACCTACCGCGAATTATTCATTTCCAGTACAT
71	ATTTTGCGTCTTTAGGAGCACTAAGCAACAGT
72	CTAAAATAGAACAAAGAAACCACCAGGGTTAG
73	GCCACGCTATACGTGGCACAGACAACGCTCAT
74	GCGTAAGAGAGAGCCAGCAGCAAAAAGGTTAT
75	GGAAATACCTACATTTTGACGCTCACCTGAAA
76	TATCACCGTACTCAGGAGGTTTAGCGGGGTTT
77	TGCTCAGTCAGTCTCTGAATTTACCAGGAGGT
78	GGAAAGCGACCAGGCGGATAAGTGAATAGGTG
79	TGAGGCAGGCGTCAGACTGTAGCGTAGCAAGG
80	TGCCTTTAGTCAGACGATTGGCCTGCCAGAAT
81	CCGGAAACACACCACGGAATAAGTAAGACTCC
82	ACGCAAAGGTCACCAATGAAACCAATCAAGTT
83	TTATTACGGTCAGAGGGTAATTGAATAGCAGC
84	TGAACAAACAGTATGTTAGCAAACTAAAAGAA
85	CTTTACAGTTAGCGAACCTCCCGACGTAGGAA
86	GAGGCGTTAGAGAATAACATAAAAGAACACCC
87	TCATTACCCGACAATAAACAACATATTTAGGC
88	CCAGACGAGCGCCCAATAGCAAGCAAGAACGC
89	AGAGGCATAATTTCATCTTCTGACTATAACTA
90	TTTTAGTTTTTCGAGCCAGTAATAAATTCTGT
91	TATGTAAACCTTTTTTAATGGAAAAATTACCT
92	TTGAATTATGCTGATGCAAATCCACAAATATA
93	GAGCAAAAACTTCTGAATAATGGAAGAAGGAG
94	TGGATTATGAAGATGATGAAACAAAATTTCAT
95	CGGAATTATTGAAAGGAATTGAGGTGAAAAAT
96	ATCAACAGTCATCATATTCCTGATTGATTGTT
97	CTAAAGCAAGATAGAACCCTTCTGAATCGTCT
98	GCCAACAGTCACCTTGCTGAACCTGTTGGCAA
99	GAAATGGATTATTTACATTGGCAGACATTCTG
100	TTTTTATAAGTATAGCCCGGCCGTCGAG
101	AGGGTTGATTTTATAAATCCTCATTAAATGATATTC
102	ACAAACAATTTTAATCAGTAGCGACAGATCGATAGC
103	AGCACCGTTTTTTAAAGGTGGCAACATAGTAGAAAA
104	TACATACATTTTGACGGGAGAATTAACTACAGGGAA
105	GCGCATTATTTTGCTTATCCGGTATTCTAAATCAGA
106	TATAGAAGTTTTCGACAAAAGGTAAAGTAGAGAATA
107	TAAAGTACTTTTCGCGAGAAAACTTTTTATCGCAAG
108	ACAAAGAATTTTATTAATTACATTTAACACATCAAG
109	AAAACAAATTTTTTCATCAATATAATCCTATCAGAT
110	GATGGCAATTTTAATCAATATCTGGTCACAAATATC
111	AAACCCTCTTTTACCAGTAATAAAAGGGATTCACCAGTCACACGTTTT
112	CCGAAATCCGAAAATCCTGTTTGAAGCCGGAA
113	CCAGCAGGGGCAAAATCCCTTATAAAGCCGGC
114	GCATAAAGTTCCACACAACATACGAAGCGCCA
115	GCTCACAATGTAAAGCCTGGGGTGGGTTTGCC
116	TTCGCCATTGCCGGAAACCAGGCATTAAATCA
117	GCTTCTGGTCAGGCTGCGCAACTGTGTTATCC
118	GTTAAAATTTTAACCAATAGGAACCCGGCACC
119	AGACAGTCATTCAAAAGGGTGAGAAGCTATAT
120	AGGTAAAGAAATCACCATCAATATAATATTTT
121	TTTCATTTGGTCAATAACCTGTTTATATCGCG
122	TCGCAAATGGGGCGCGAGCTGAAATAATGTGT
123	TTTTAATTGCCCGAAAGACTTCAAAACACTAT
124	AAGAGGAACGAGCTTCAAAGCGAAGATACATT
125	GGAATTACTCGTTTACCAGACGACAAAAGATT
126	GAATAAGGACGTAACAAAGCTGCTCTAAAACA
127	CCAAATCACTTGCCCTGACGAGAACGCCAAAA
128	CTCATCTTGAGGCAAAAGAATACAGTGAATTT
129	AAACGAAATGACCCCCAGCGATTATTCATTAC
130	CTTAAACATCAGCTTGCTTTCGAGCGTAACAC
131	TCGGTTTAGCTTGATACCGATAGTCCAACCTA
132	TGAGTTTCGTCACCAGTACAAACTTAATTGTA
133	CCCCGATTTAGAGCTTGACGGGGAAATCAAAA
134	GAATAGCCGCAAGCGGTCCACGCTCCTAATGA
135	GAGTTGCACGAGATAGGGTTGAGTAAGGGAGC
136	GTGAGCTAGTTTCCTGTGTGAAATTTGGGAAG
137	TCATAGCTACTCACATTAATTGCGCCCTGAGA
138	GGCGATCGCACTCCAGCCAGCTTTGCCATCAA
139	GAAGATCGGTGCGGGCCTCTTCGCAATCATGG
140	AAATAATTTTAAATTGTAAACGTTGATATTCA
141	GCAAATATCGCGTCTGGCCTTCCTGGCCTCAG
142	ACCGTTCTAAATGCAATGCCTGAGAGGTGGCA
143	TATATTTTAGCTGATAAATTAATGTTGTATAA
144	TCAATTCTTTTAGTTTGACCATTACCAGACCG
145	CGAGTAGAACTAATAGTAGTAGCAAACCCTCA
146	GAAGCAAAAAAGCGGATTGCATCAGATAAAAA
147	TCAGAAGCCTCCAACAGGTCAGGATCTGCGAA
148	CCAAAATATAATGCAGATACATAAACACCAGA
149	CATTCAACGCGAGAGGCTTTTGCATATTATAG
150	ACGAGTAGTGACAAGAACCGGATATACCAAGC
151	AGTAATCTTAAATTGGGCTTGAGAGAATACCA
152	GCGAAACATGCCACTACGAAGGCATGCGCCGA
153	ATACGTAAAAGTACAACGGAGATTTCATCAAG
154	CAATGACACTCCAAAAGGAGCCTTACAACGCC
155	AAAAAAGGACAACCATCGCCCACGCGGGTAAA

156	TGTAGCATTCCACAGACAGCCCTCATCTCCAA
157	GTAAAGCACTAAATCGGAACCCTAGTTGTTCC
158	AGTTTGGAGCCCTTCACCGCCTGGTTGCGCTC
159	AGCTGATTACAAGAGTCCACTATTGAGGTGCC
160	ACTGCCCGCCGAGCTCGAATTCGTTATTACGC
161	CCCGGGTACTTTCCAGTCGGGAAACGGGCAAC
162	CAGCTGGCGGACGACGACAGTATCGTAGCCAG
163	GTTTGAGGGAAAGGGGGATGTGCTAGAGGATC
164	CTTTCATCCCCAAAAACAGGAAGACCGGAGAG
165	AGAAAAGCAACATTAAATGTGAGCATCTGCCA
166	GGTAGCTAGGATAAAAATTTTTAGTTAACATC
167	CAACGCAATTTTTGAGAGATCTACTGATAATC
168	CAATAAATACAGTTGATTCCCAATTTAGAGAG
169	TCCATATACATACAGGCAAGGCAACTTTATTT
170	TACCTTTAAGGTCTTTACCCTGACAAAGAAGT
171	CAAAAATCATTGCTCCTTTTGATAAGTTTCAT
172	TTTGCCAGATCAGTTGAGATTTAGTGGTTTAA
173	AAAGATTCAGGGGGTAATAGTAAACCATAAAT
174	TTTCAACTATAGGCTGGCTGACCTTGTATCAT
175	CCAGGCGCTTAATCATTGTGAATTACAGGTAG
176	CGCCTGATGGAAGTTTCCATTAAACATAACCG
177	TTTCATGAAAATTGTGTCGAAATCTGTACAGA
178	ATATATTCTTTTTTCACGTTGAAAATAGTTAG
179	AATAATAAGGTCGCTGAGGCTTGCAAAGACTT
180	CGTAACGATCTAAAGTTTTGTCGTGAATTGCG
181	ACCCAAATCAAGTTTTTTGGGGTCAAAGAACG
182	TGGACTCCCTTTTCACCAGTGAGACCTGTCGT
183	TGGTTTTTAACGTCAAAGGGCGAAGAACCATC
184	GCCAGCTGCCTGCAGGTCGACTCTGCAAGGCG
185	CTTGCATGCATTAATGAATCGGCCCGCCAGGG
186	ATTAAGTTCGCATCGTAACCGTGCGAGTAACA
187	TAGATGGGGGGTAACGCCAGGGTTGTGCCAAG
188	ACCCGTCGTCATATGTACCCCGGTAAAGGCTA
189	CATGTCAAGATTCTCCGTGGGAACCGTTGGTG
190	TCAGGTCACTTTTGCGGGAGAAGCAGAATTAG
191	CTGTAATATTGCCTGAGAGTCTGGAAAACTAG
192	CAAAATTAAAGTACGGTGTCTGGAAGAGGTCA
193	TGCAACTAAGCAATAAAGCCTCAGTTATGACC
194	TTTTTGCGCAGAAAACGAGAATGAATGTTTAG
195	AAACAGTTGATGGCTTAGAGCTTATTTAAATA
196	ACTGGATAACGGAACAACATTATTACCTTATG
197	ACGAACTAGCGTCCAATACTGCGGAATGCTTT
198	CGATTTTAGAGGACAGATGAACGGCGCGACCT
199	CTTTGAAAAGAACTGGCTCATTATTTAATAAA
200	GCTCCATGAGAGGCTTTGAGGACTAGGGAGTT
201	ACGGCTACTTACTTAGCCGGAACGCTGACCAA
202	AAAGGCCGAAAGGAACAACTAAAGCTTTCCAG
203	GAGAATAGCTTTTGCGGGATCGTCGGGTAGCA
204	ACGTTAGTAAATGAATTTTCTGTAAGCGGAGT
205	TTTTCGATGGCCCACTACGTAAACCGTC
206	TATCAGGGTTTTCGGTTTGCGTATTGGGAACGCGCG
207	GGGAGAGGTTTTTGTAAAACGACGGCCATTCCCAGT
208	CACGACGTTTTTGTAATGGGATAGGTCAAAACGGCG
209	GATTGACCTTTTGATGAACGGTAATCGTAGCAAACA
210	AGAGAATCTTTTGGTTGTACCAAAAACAAGCATAAA
211	GCTAAATCTTTTCTGTAGCTCAACATGTATTGCTGA
212	ATATAATGTTTTCATTGAATCCCCCTCAAATCGTCA
213	TAAATATTTTTTGGAAGAAAAATCTACGACCAGTCA
214	GGACGTTGTTTTTCATAAGGGAACCGAAAGGCGCAG
215	ACGGTCAATTTTGACAGCATCGGAACGAACCCTCAG
216	CAGCGAAATTTTAACTTTCAACAGTTTCTGGGATTTTGCTAAACTTTT

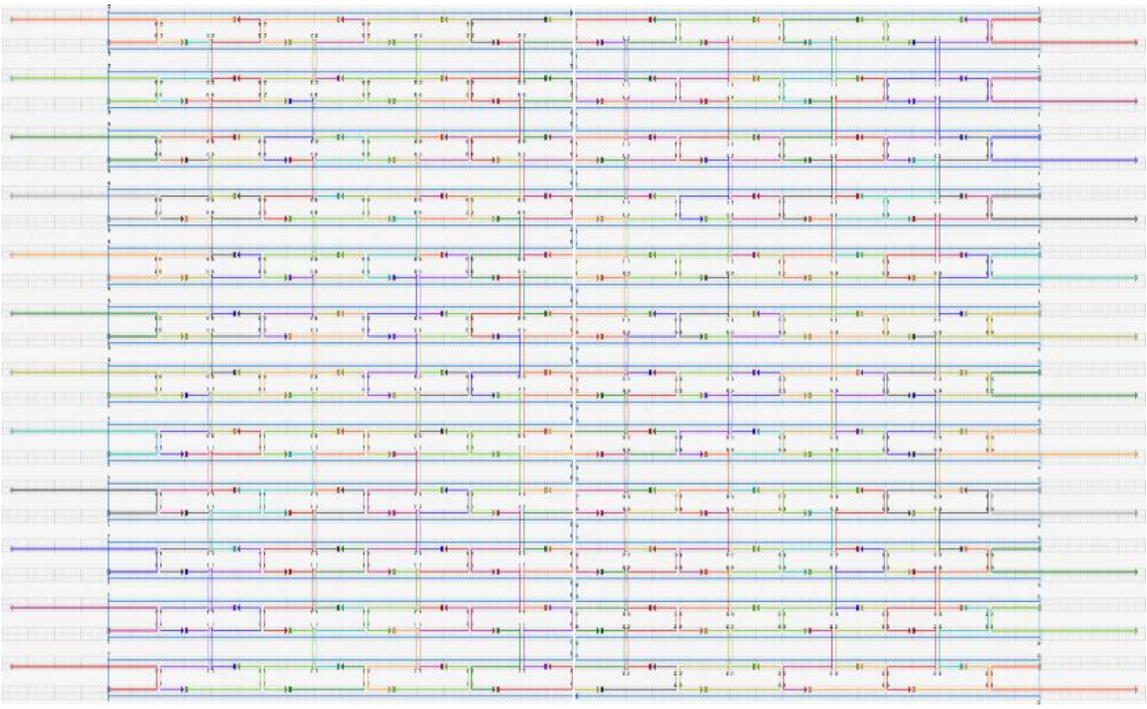

Figure S7. Origami with poly-dA design. To add the poly-dA_{30} overhangs, staple strands 100-111 and 205-216 were removed from the design and replaced with staple strands with dA30 at their 3' ends. The new staple sequences are listed in Table S3.

Table S3. Staple strand sequences modified for the origami with poly-dA.

100-30A	TATAAGTATAGCCCGGCCGTCGAGAGGGTTGAAAAAAAAAAAAAAAAAAAAAAAAAAAAAA
101-30A	ATAAATCCTCATTAAATGATATTCACAAACAAAAAAAAAAAAAAAAAAAAAAAAAAAAAA
102-30A	AATCAGTAGCGACAGATCGATAGCAGCACCGTAAAAAAAAAAAAAAAAAAAAAAAAAAAAAA
103-30A	TAAAGGTGGCAACATAGTAGAAAATACATACAAAAAAAAAAAAAAAAAAAAAAAAAAAAAA
104-30A	GACGGGAGAATTAACTACAGGGAAGCGCATTAAAAAAAAAAAAAAAAAAAAAAAAAAAAAA
105-30A	GCTTATCCGGTATTCTAAATCAGATATAGAAGAAAAAAAAAAAAAAAAAAAAAAAAAAAAAA
106-30A	CGACAAAAGGTAAAGTAGAGAATATAAAGTACAAAAAAAAAAAAAAAAAAAAAAAAAAAAAA
107-30A	CGCGAGAAAACTTTTTATCGCAAGACAAAGAAAAAAAAAAAAAAAAAAAAAAAAAAAAAA
108-30A	ATTAATTACATTTAACACATCAAGAAAACAAAAAAAAAAAAAAAAAAAAAAAAAAAAAA
109-30A	TTCATCAATATAATCCTATCAGATGATGGCAAAAAAAAAAAAAAAAAAAAAAAAAAAAAA
110-30A	AATCAATATCTGGTCACAAATATCAAACCCTCAAAAAAAAAAAAAAAAAAAAAAAAAAAAAA
111-30A	ACCAGTAATAAAAGGGATTCACCAGTCACACGAAAAAAAAAAAAAAAAAAAAAAAAAAAAAA
205-30A	CGATGGCCCACTACGTAAACCGTCTATCAGGGAAAAAAAAAAAAAAAAAAAAAAAAAAAAAA
206-30A	CGGTTTGCGTATTGGGAACGCGCGGGGAGAGGAAAAAAAAAAAAAAAAAAAAAAAAAAAAAA
207-30A	TGTAAAACGACGGCCATTCCCAGTCACGACGTAAAAAAAAAAAAAAAAAAAAAAAAAAAAAA
208-30A	GTAATGGGATAGGTCAAAACGGCGGATTGACCAAAAAAAAAAAAAAAAAAAAAAAAAAAAAA
209-30A	GATGAACGGTAATCGTAGCAAACAAGAGAATCAAAAAAAAAAAAAAAAAAAAAAAAAAAAAA
210-30A	GGTTGTACCAAAAACAAGCATAAAGCTAAATCAAAAAAAAAAAAAAAAAAAAAAAAAAAAAA
211-30A	CTGTAGCTCAACATGTATTGCTGAATATAATGAAAAAAAAAAAAAAAAAAAAAAAAAAAAAA
212-30A	CATTGAATCCCCCTCAAATCGTCATAAATATTAAAAAAAAAAAAAAAAAAAAAAAAAAAAAA
213-30A	GGAAGAAAAATCTACGACCAGTCAGGACGTTGAAAAAAAAAAAAAAAAAAAAAAAAAAAAAA
214-30A	TCATAAGGGAACCGAAAGGCGCAGACGGTCAAAAAAAAAAAAAAAAAAAAAAAAAAAAAA
215-30A	GACAGCATCGGAACGAACCCTCAGCAGCGAAAAAAAAAAAAAAAAAAAAAAAAAAAAAA
216-30A	AACTTTCAACAGTTTCTGGGATTTTGCTAAACAAAAAAAAAAAAAAAAAAAAAAAAAAAAAA

To determine the concentrations of the DNA origami templates, their absorbance at 260 nm was measured by NanoDrop Lite Spectrophotometer (ThermoFisher Scientific). The extinction coefficient of each DNA origami template design can be approximated by equation (1).[44]

$$\varepsilon = 6700ds + 10000ss \quad (1)$$

where ds is the number of double stranded bases and ss is the number of single-stranded bases. Using Lambert- Beer's law (A260 nm = εbc, b = 1 cm), the concentrations of DNA origami templates can be calculated.

Table S4. Sequences used in the silencing experiments. Bases highlighted are FANA, bases underlined are phosphorotioated.[35]

Name	Sequence (5’ → 3’)
**ASO**	**AUAUC**CTTGTCGT**AUCCC**
**Scramble**	**AAUA**ATTGAATTCT**UCAA**
**ASO-Integrated**	AAAAAAAAAAAAAAAAAAAAAAAAAAAAAA**AUAUC**CTTGTCGT**AUCCC**
**Scramble-Integrated**	AAAAAAAAAAAAAAAAAAAAAAAAAAAAAA**AAUA**ATTGAATTCT**UCAA**
**ASO-Complement**	AAAAAAAAAAAAAAAAAAAAAAAAAAAAAATTACGGGATACGACAA
**Scramble-Complement**	AAAAAAAAAAAAAAAAAAAAAAAAAAAAAATTACTTGAAGAATTCAATT
**$dA_{30}C_8$**	AAAAAAAAAAAAAAAAAAAAAAAAAAAAAACCCCCCCC

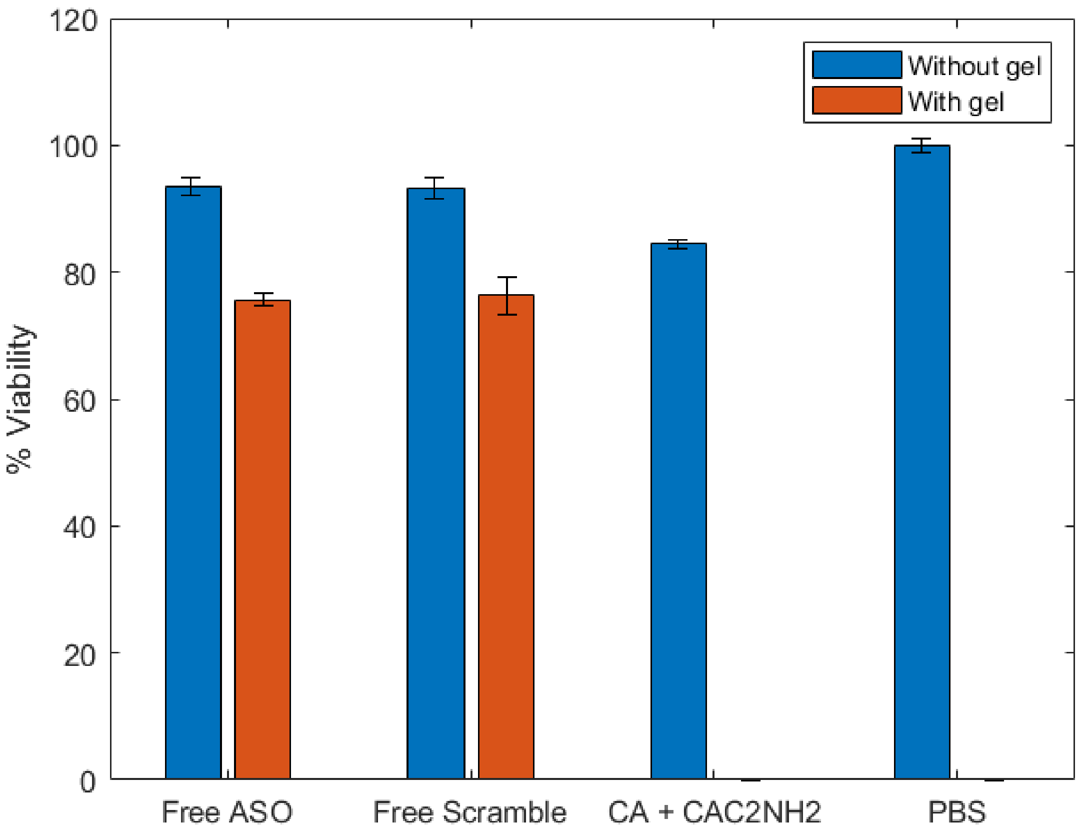

Figure S8. Cell viability comparison with and without the presence of the hydrogel in the 24h experiment. Free scramble and free ASO without gel are in a PBS buffer. CA + CAC2NH2

column is with 20mM of CA, 30mM of $CAC2NH_2$ in Serum buffer, like the hydrogel environment. Error bars represent one standard deviation.

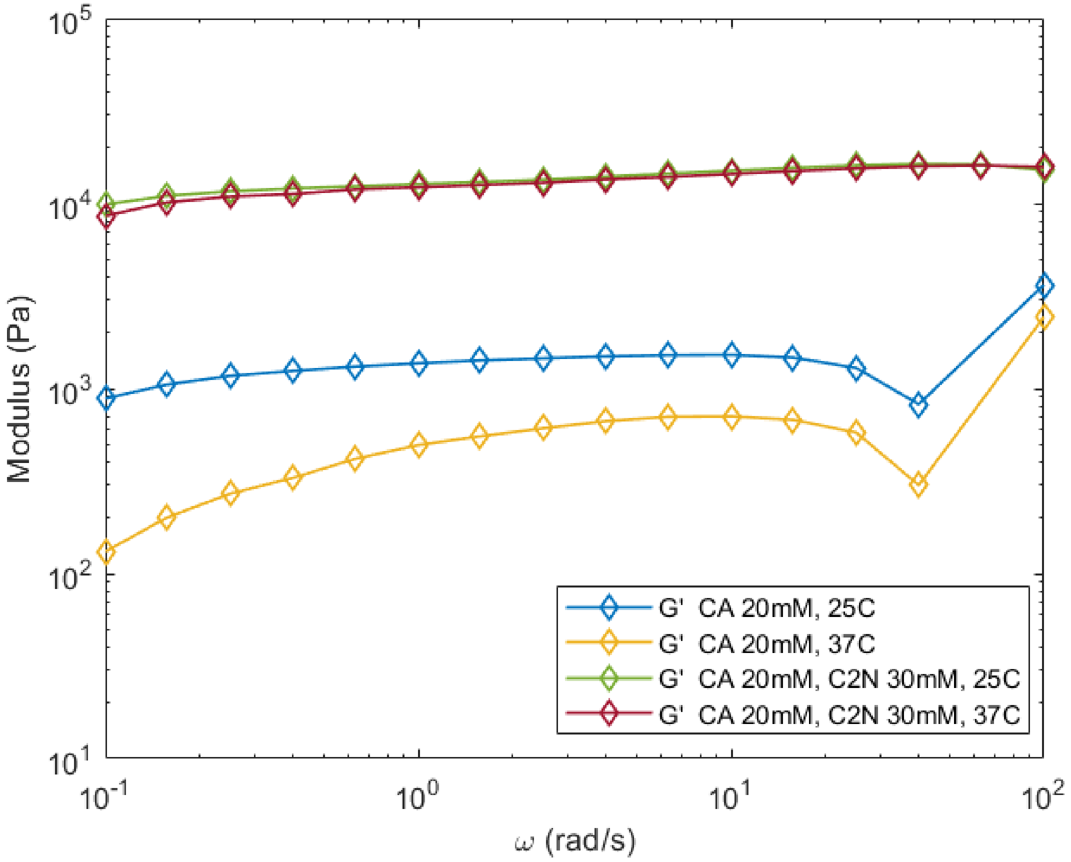

Figure S9. Thermal stability of rheological properties of $dA_{30}C_8$ 700µM , CA 20mM, $CAC2NH_2$ 30mM, Serum buffer from room temperature to 37˚C.

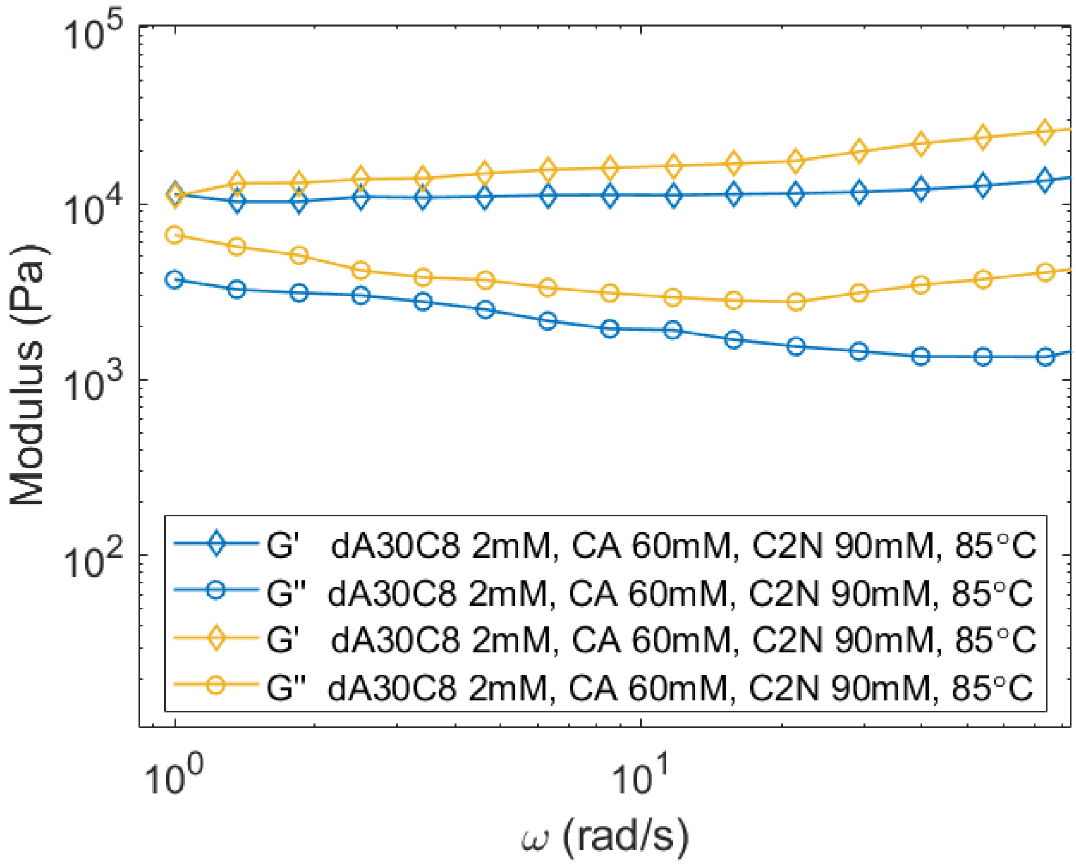

Figure S10. Thermal stability of rheological properties of duplicates of $dA_{30}C_8$ 2000μM , CA 60mM, $CAC2NH_2$ 90mM, Serum buffer at 85˚C.

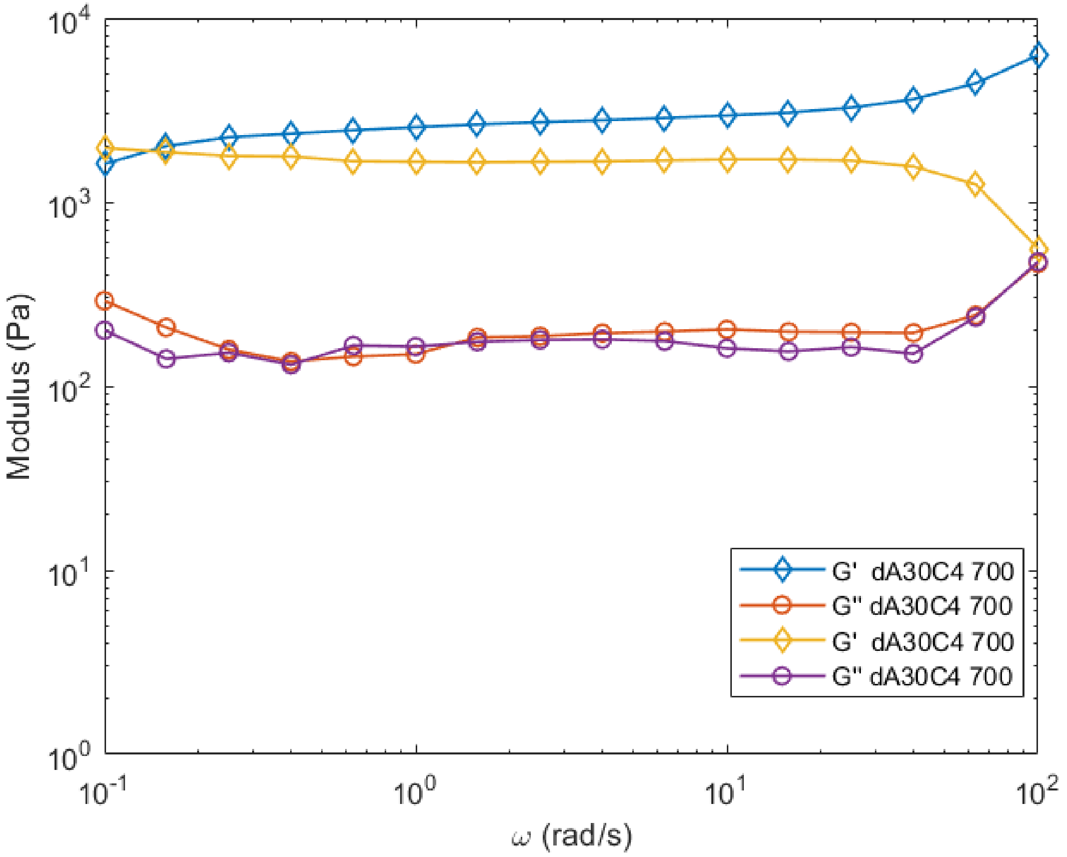

Figure S11. Replicates of $dA_{30}C_4$ 700µM , CA 20mM Mag buffer at 25˚C.

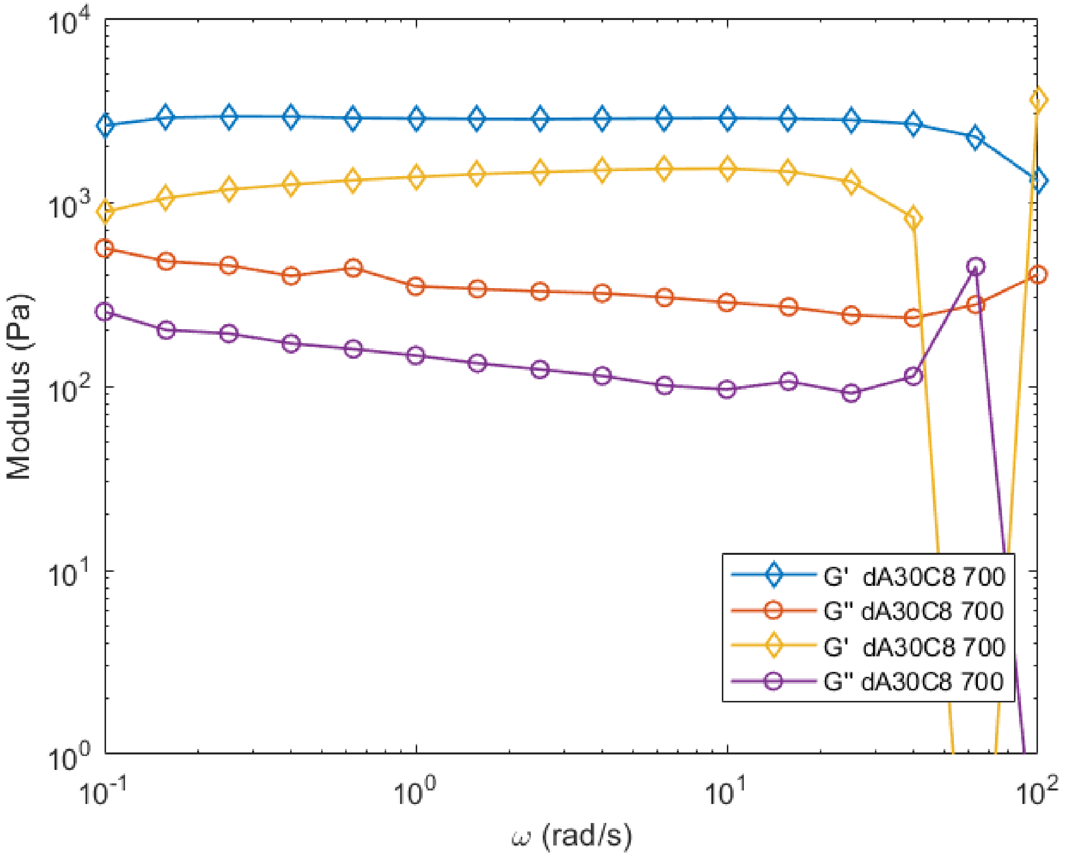

Figure S12. Replicates of $dA_{30}C_8$ 700µM , CA 20mM Mag buffer at 25˚C.

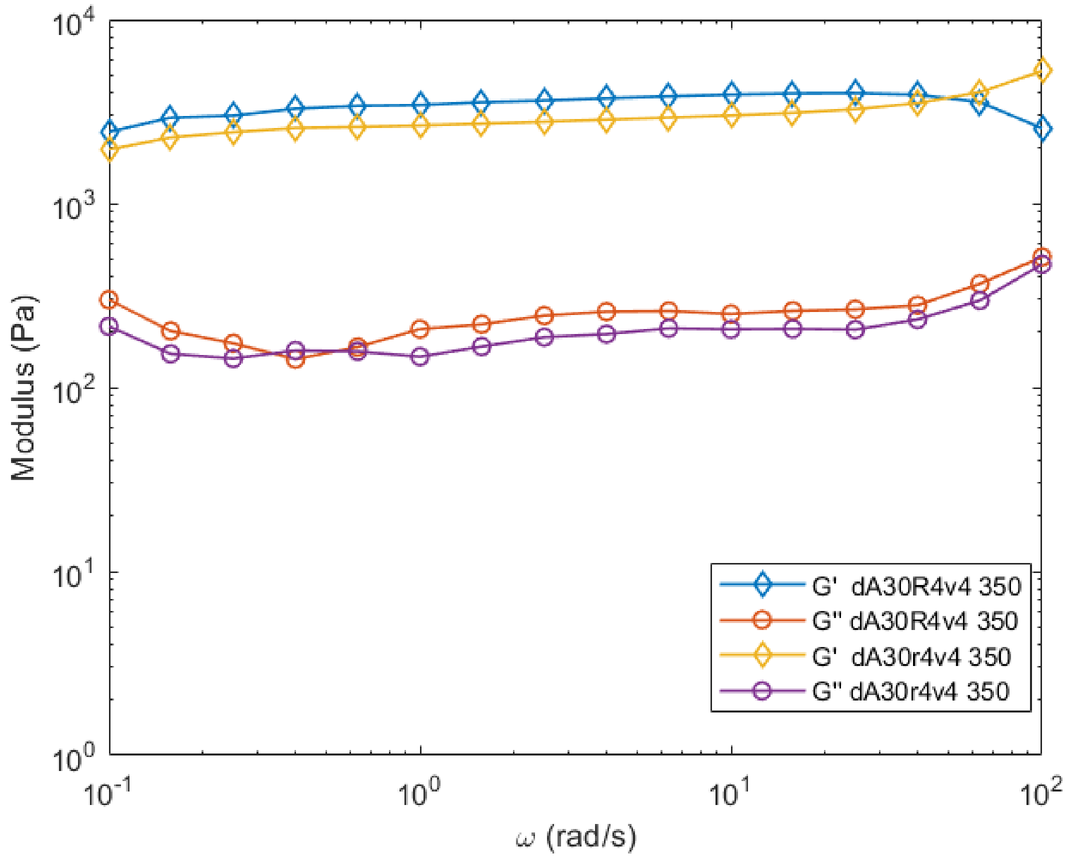

Figure S13. Replicates of dA_{30}-dsDNA13 700μM , CA 20mM Mag buffer at 25˚C.

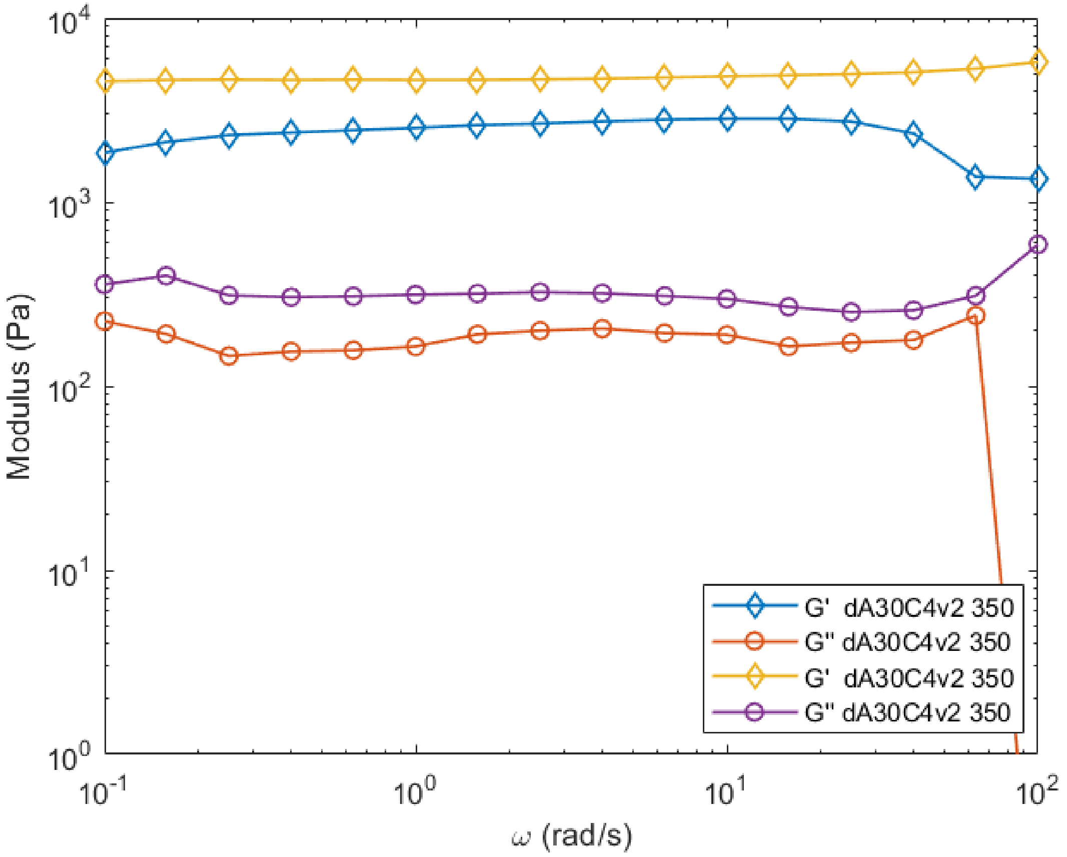

Figure S14. Replicates of dA_{30}-dsDNA20 700µM , CA 20mM Mag buffer at 25˚C.

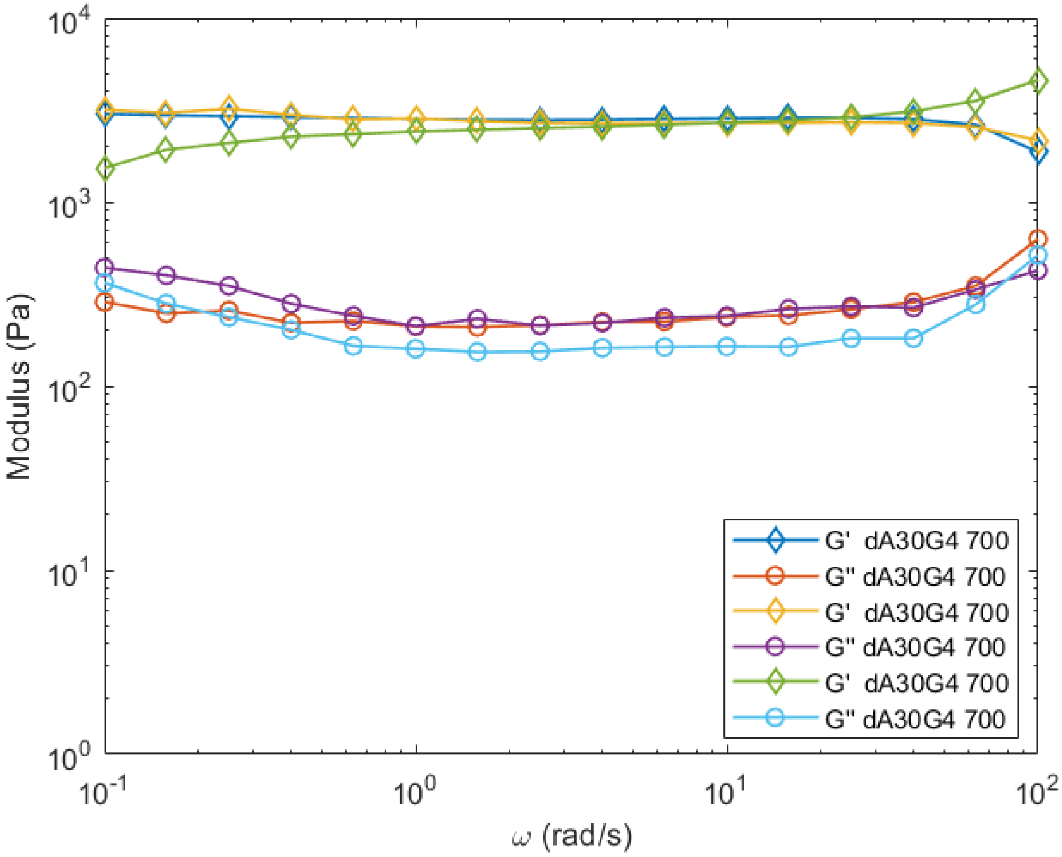

Figure S15. Replicates of $dA_{30}G_4$ 700µM , CA 20mM Mag buffer at 25˚C.

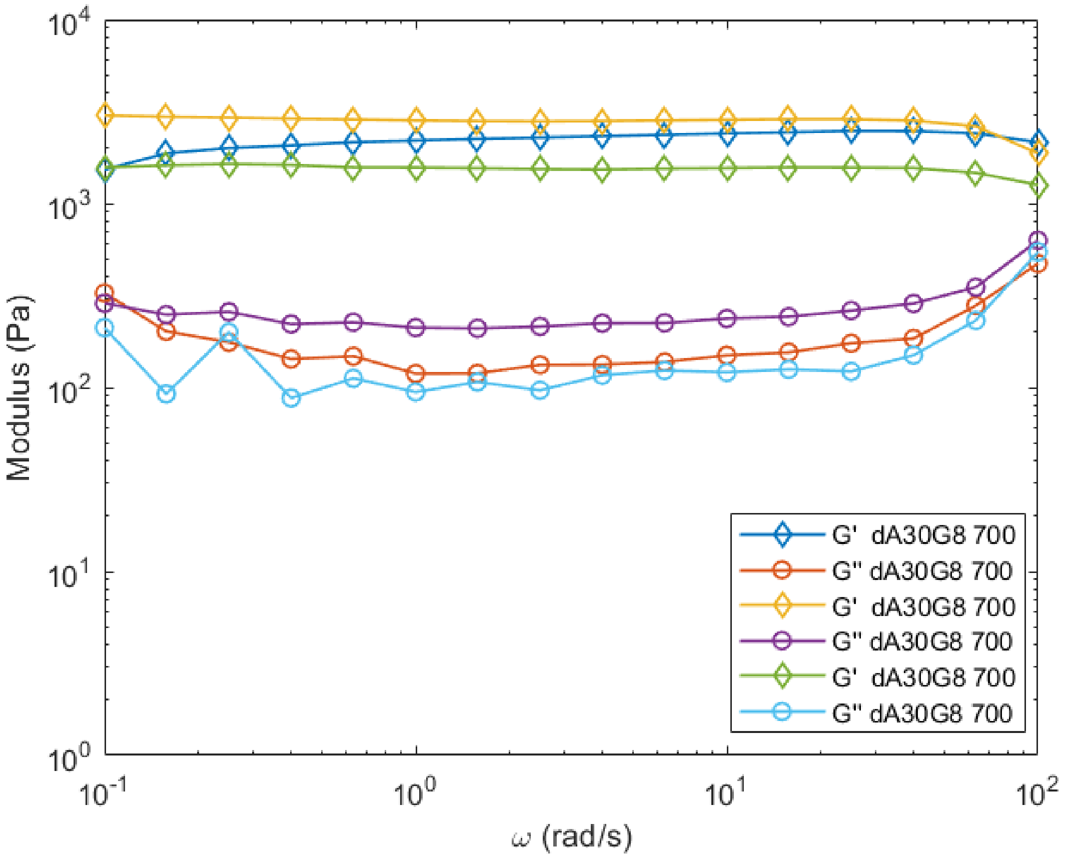

Figure S16. Replicates of $dA_{30}G_8$ 700µM , CA 20mM Mag buffer at 25˚C.

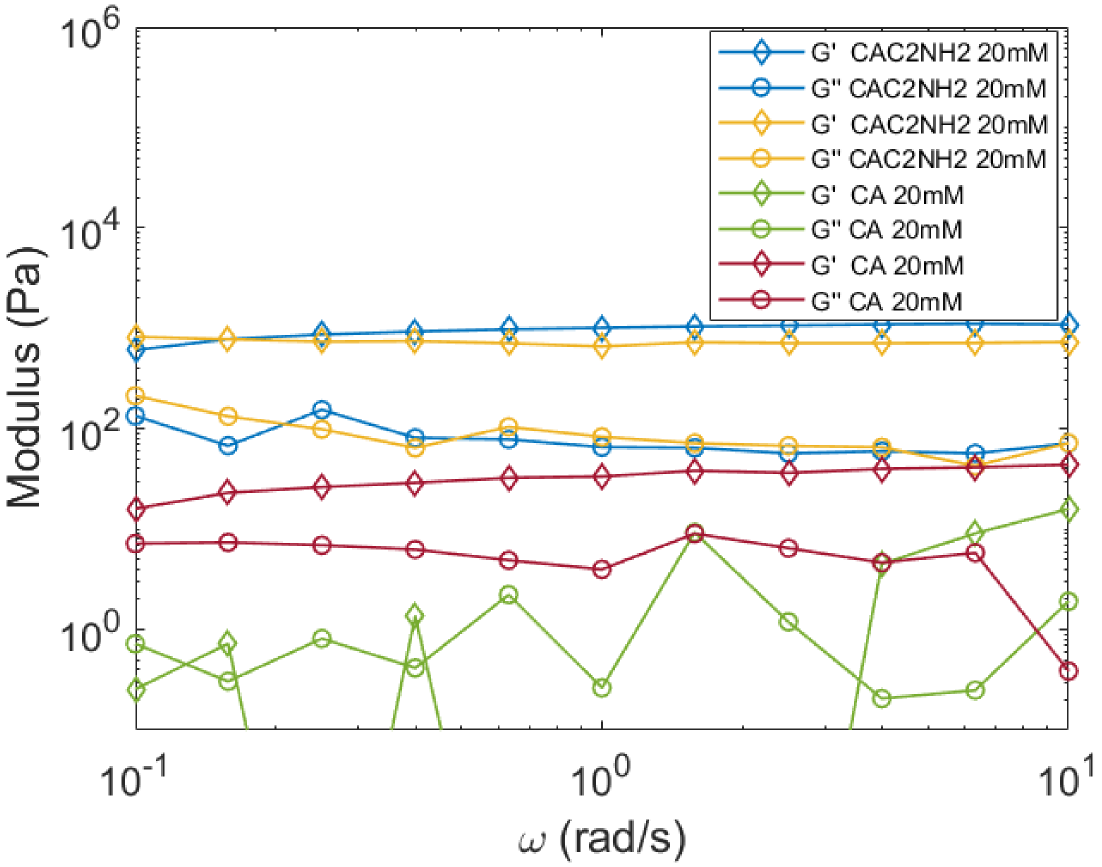

Figure S17. Replicates of dA_{15} 700µM , CA or $CAC2NH_2$ 20mM Mag buffer at 25˚C. The first replicate of CA 20mM shows some slippage, the values used for the moduli at 1Hz are interpolated linearly.

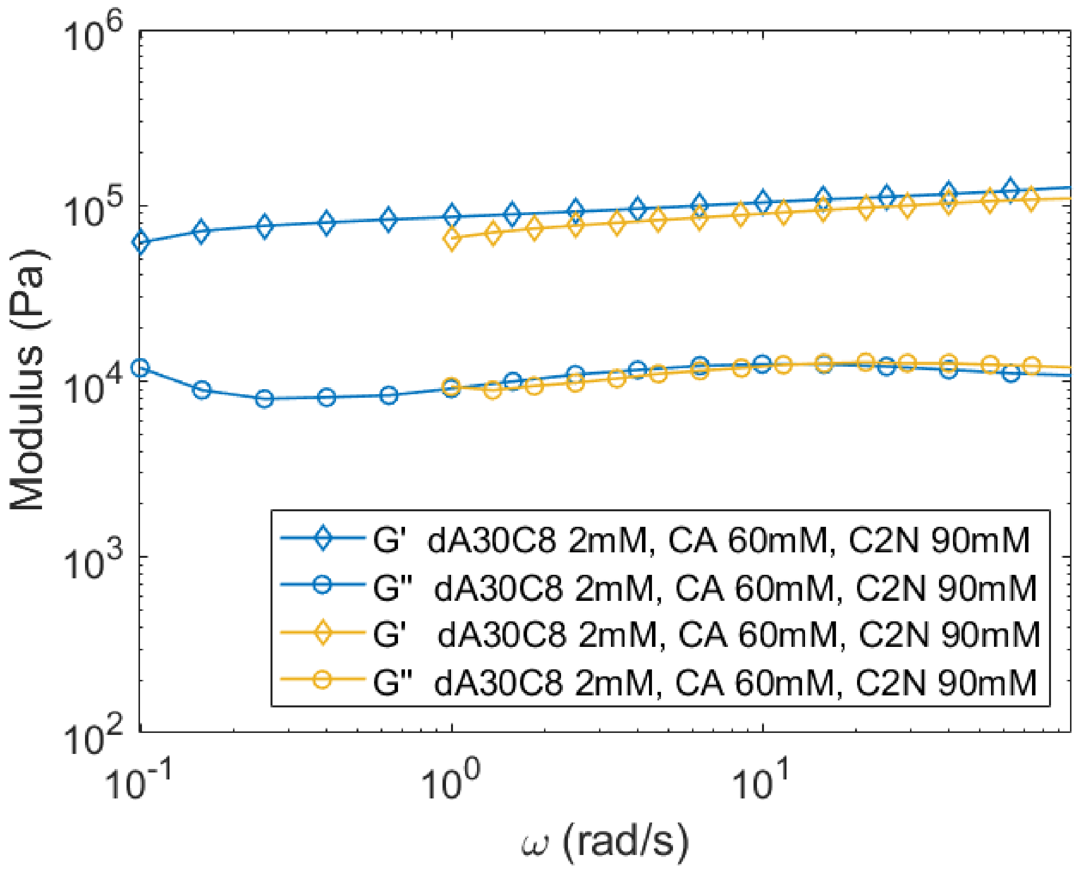

Figure S18. Replicates of $dA_{30}C_8$ 700µM , CA 60mM, $CAC2NH_2$ 90mM Serum buffer at 25˚C. Value at 10Hz for the second replicate is interpolated linearly for the calculation of the mean presented in the manuscript.

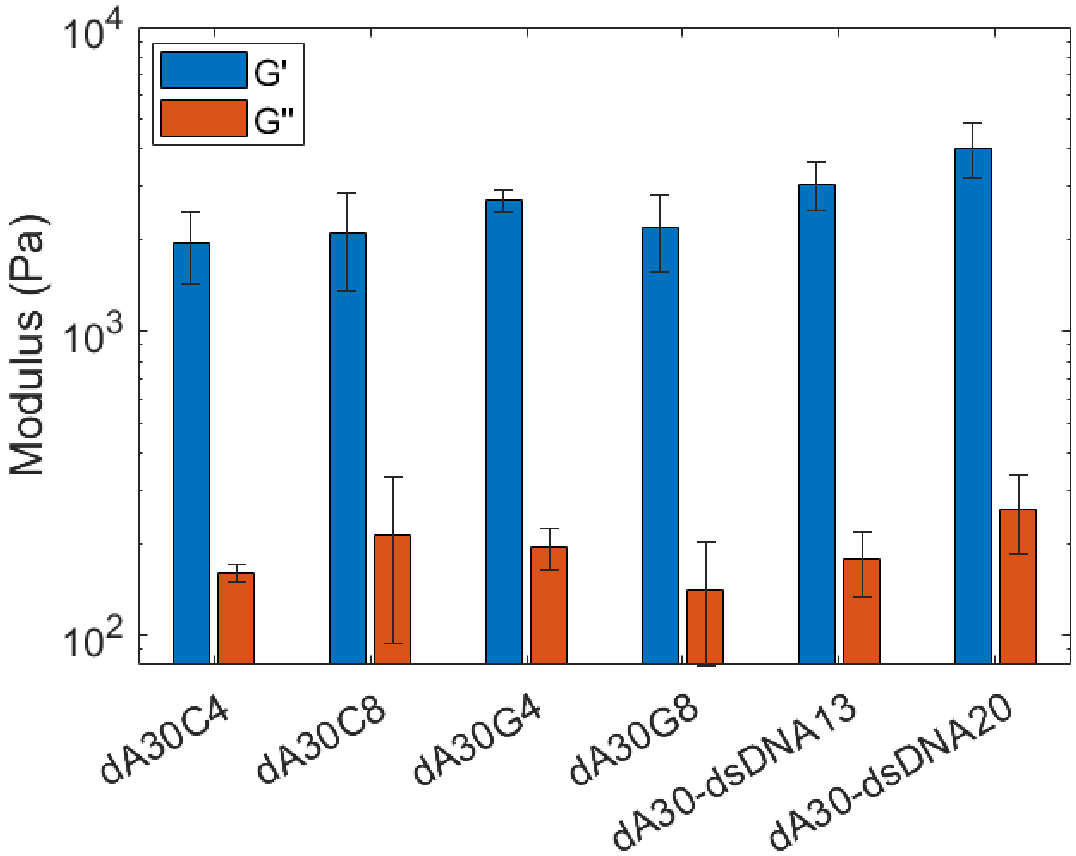

Figure S19. Mean of moduli of dA_{30} with different crosslinking regions at 700µM, CA 20mM and Mag buffer at 25˚C, 1Hz.

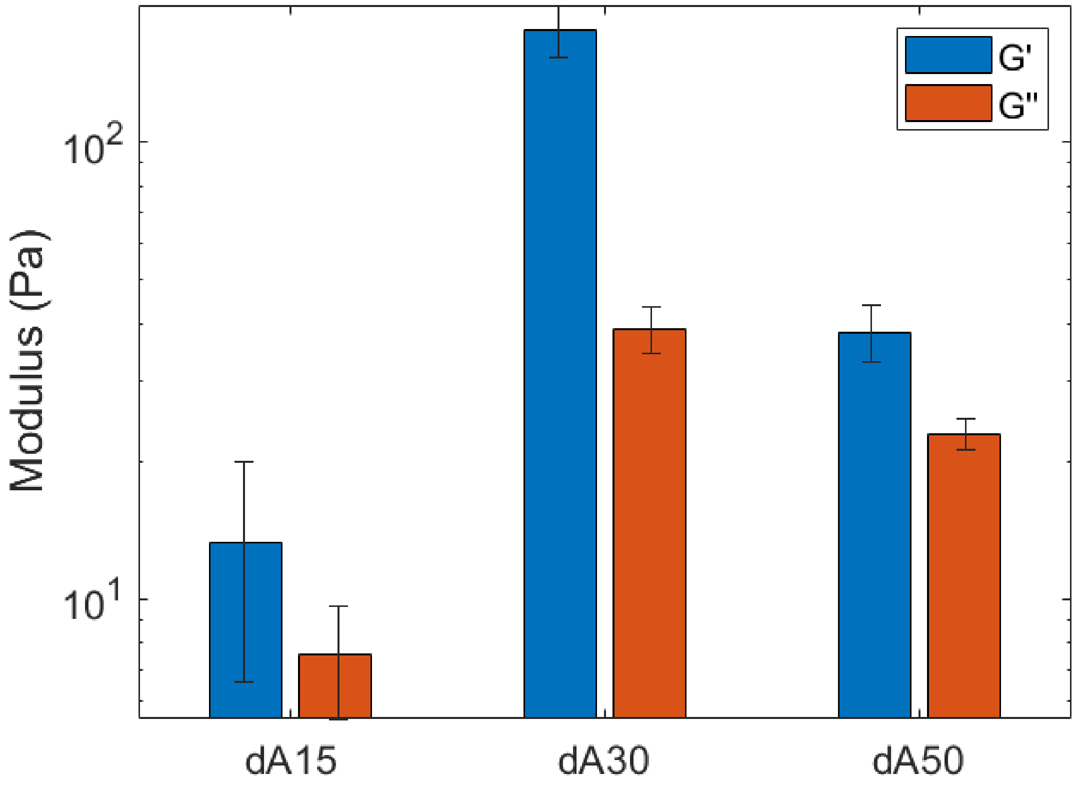

Figure S20. Replicates of dA_{15} (1400µM), dA_{30} (700µM) and dA_{50} (420µM) CA 20mM Mag buffer at 25˚C, 1Hz.

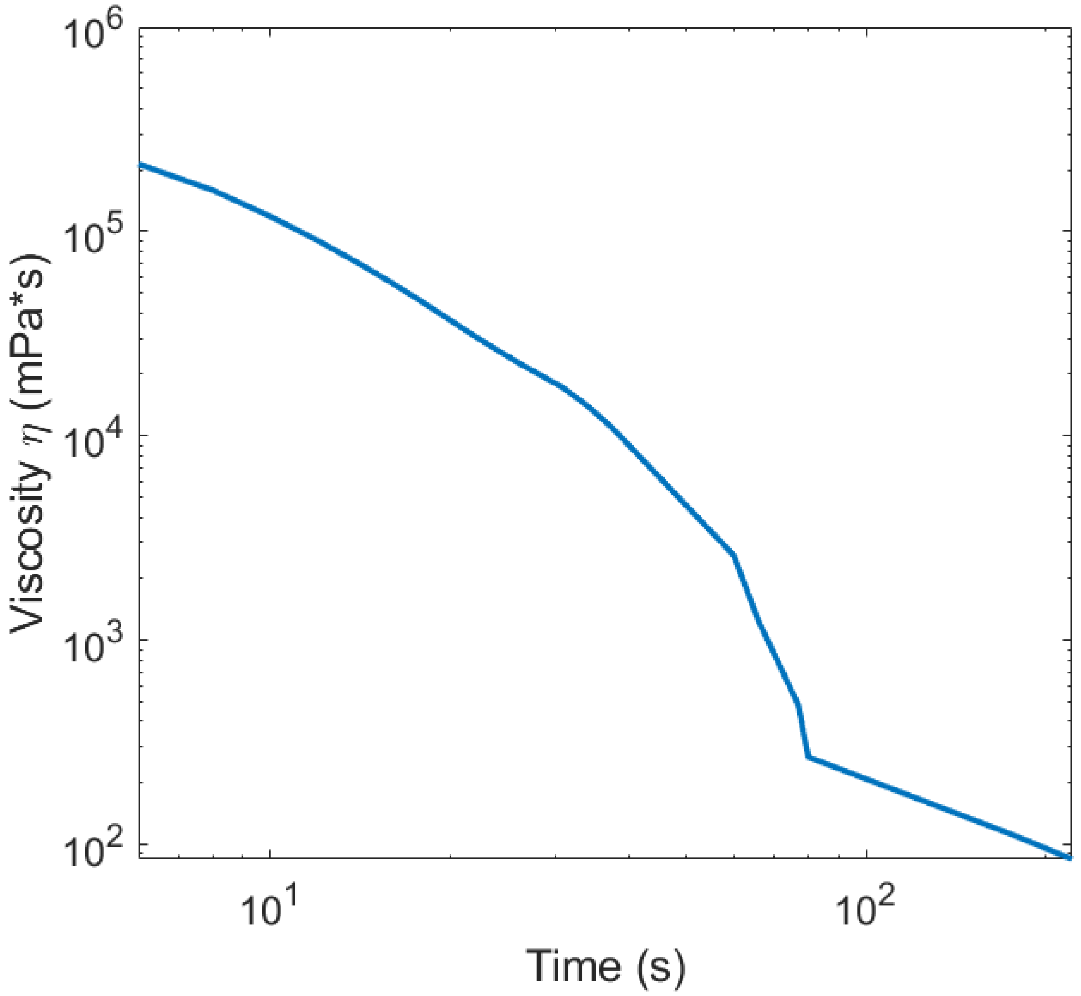

Figure S21. Viscosity versus time in the Shear thinning experiment of Figure 3d (hydrogel $dA_{30}C_4$ 700µM, CA 20mM, Mag buffer).

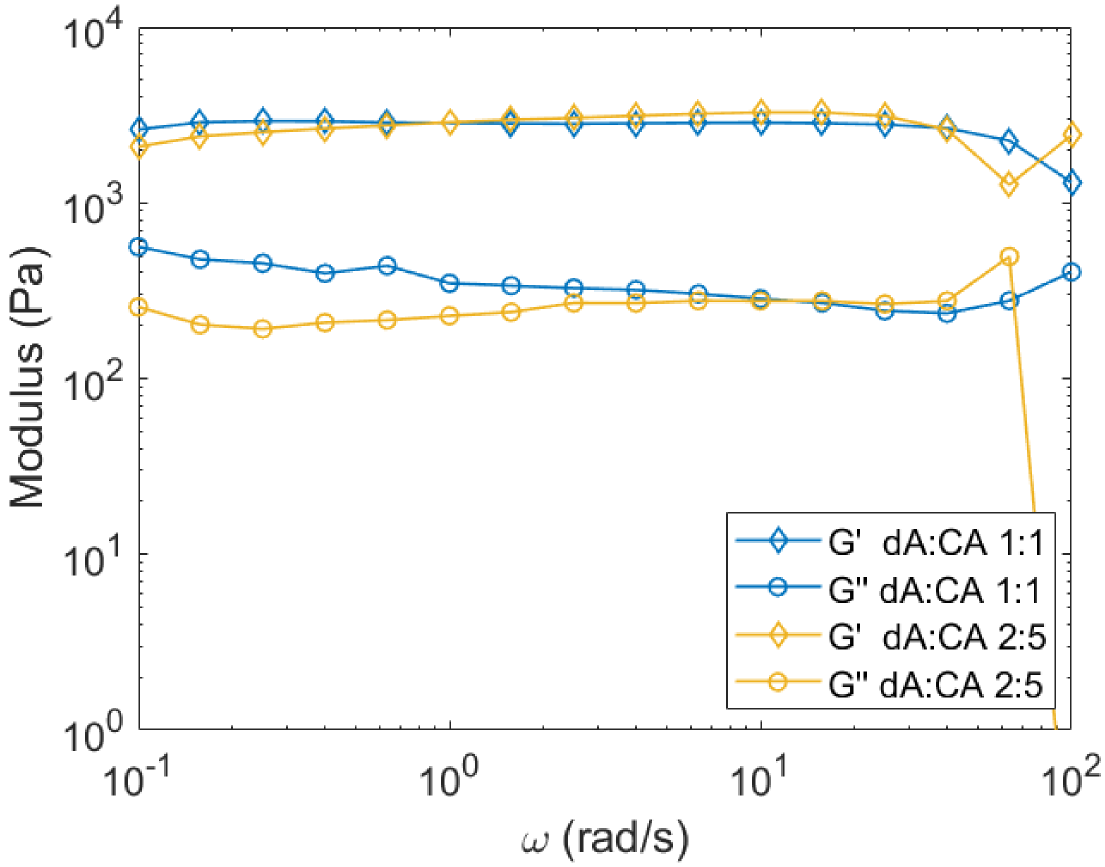

Figure S23. Variation in stoechoiometric ratios for $dA_{30}C_8$ 700μM Serum buffer at 25˚C, showing a similar value when there is excess CA for dA.

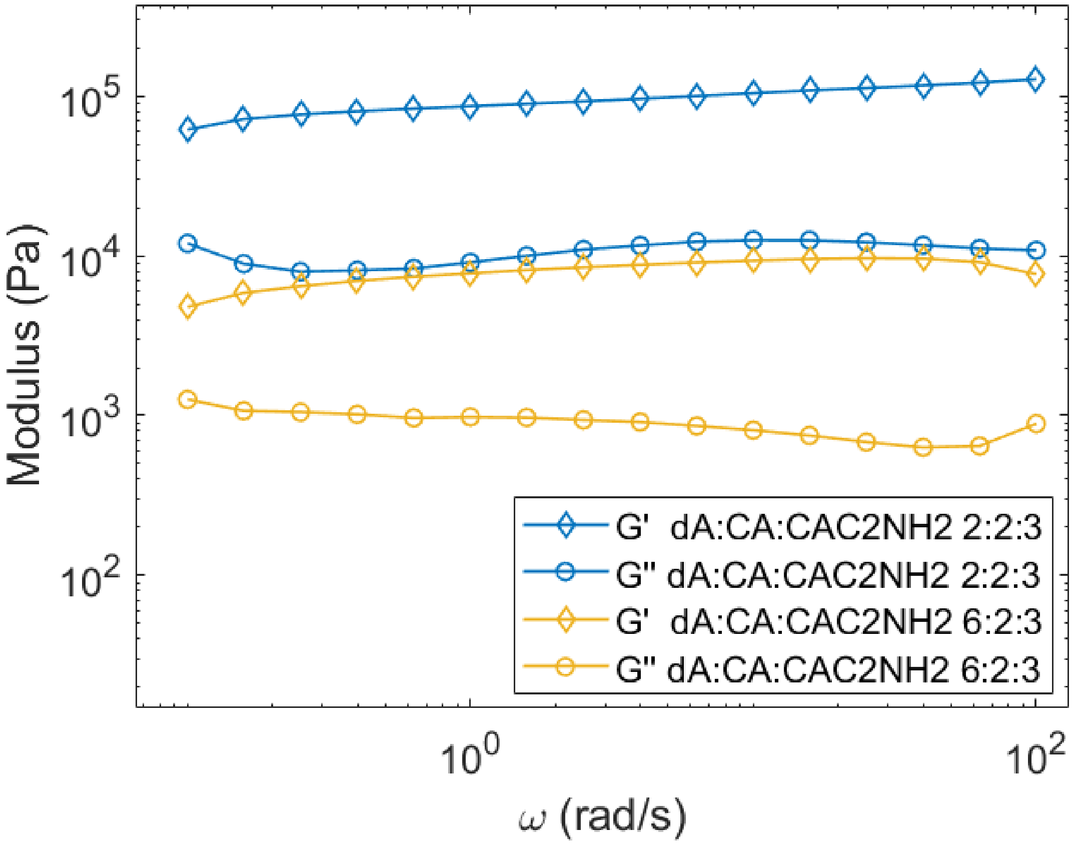

Figure S23. Variation in stoechoiometric ratios for $dA_{30}C_8$ 2mM Serum buffer at 25˚C, showing a lower than potential value when there is excess dA for CA:CAC2NH2.
